# Functional Profile of γδ T Cells in Severe and Moderate COVID-19: A Brazilian Cross-Sectional Study

**DOI:** 10.3390/cells15111020

**Published:** 2026-06-01

**Authors:** Andressa da Silva Cazote, Glenda Domingos Mascarenhas, Hugo Perazzo, Kim Mattos Geraldo, Maria Pia Diniz Ribeiro, Juliana Arruda de Matos, Pedro Emmanuel Alvarenga Americano do Brasil, Sandra Wagner Cardoso, Beatriz Grinsztejn, Valdiléa Gonçalves Veloso, Cynthia Machado Cascabulho, José Henrique Pilotto, Diogo Gama Caetano, Milena Neira Guimarães Goulart, Nathalia Beatriz Ramos de Sá, Dalziza Victalina de Almeida, Fernanda Heloise Côrtes, Mariza Gonçalves Morgado, Carmem Beatriz Wagner Giacoia-Gripp

**Affiliations:** 1 Laboratório de AIDS & Imunologia Molecular, Instituto Oswaldo Cruz (IOC), FIOCRUZ, Rio de Janeiro 21040-360, Brazil; andressacazote@gmail.com (A.d.S.C.); gdomingos@biof.ufrj.br (G.D.M.); pilotto@ioc.fiocruz.br (J.H.P.); diogo.caetano91@gmail.com (D.G.C.); milenanggoulart@gmail.com (M.N.G.G.); nathalia.ramos@ioc.fiocruz.br (N.B.R.d.S.); dalziza@ioc.fiocruz.br (D.V.d.A.); fheloise@ioc.fiocruz.br (F.H.C.); mmorgado@ioc.fiocruz.br (M.G.M.); 2 Instituto Nacional de Infectologia Evandro Chagas (INI), FIOCRUZ, Rio de Janeiro 21040-360, Brazil; perazzohugo@gmail.com (H.P.); kim.geraldo@ini.fiocruz.br (K.M.G.); mariapia.diniz@ini.fiocruz.br (M.P.D.R.); juliana.matos@ini.fiocruz.br (J.A.d.M.); pedro.brasil@fiocruz.br (P.E.A.A.d.B.); sandra.wagner@ini.fiocruz.br (S.W.C.); beatriz.grinsztejn@gmail.com (B.G.); valdilea.veloso@gmail.com (V.G.V.); 3 Flow Cytometry Platform—Multiparametric Analysis Unit, Oswaldo Cruz Institute, Rio de Janeiro 21040-360, Brazil; cynthiac@ioc.fiocruz.br

**Keywords:** COVID-19, γδ T cells, disease severity

## Abstract

This study aimed to identify the distinct intrinsic response potential of γδ T cells from COVID-19 patients with different illness severities, to better understand the implication of these cells in COVID-19 disease. Forty-four COVID patients were enrolled at hospitalization and classified as: moderate without oxygen support (MWO_2_; N = 15), moderate with oxygen support (MO_2_; N = 15), or severe disease requiring mechanical ventilation (SD; N = 14). γδ T cells were characterized ex vivo, isolated from peripheral blood cells, stimulated in vitro with OKT3 and K562 cells, and evaluated for functional markers by flow cytometry. Ex vivo analysis identified 16.21% of total γδ T cells as Vδ1^−^Vδ2^−^. SD patients presented a lower frequency of TRAIL^+^ and of IL-17-producing Vδ2 cells, as well as lower value of fluorescence intensity values for TNF-α in Vδ2 cells, than MWO_2_ patients (*p* < 0.05). In addition, paired analyses showed a lower frequency of IL-17-producing than CD161^+^ Vδ2 cells in SD patients (*p* < 0.05). These observations suggest a more restricted response potential of the Vδ2 subset in severe disease, show the impact of general immune dysregulation on these cells, or even suggest some role for IL-17-producing Vδ2 cells in preventing critical symptoms.

## 1. Introduction

Coronavirus disease 2019 (COVID-19), caused by the severe acute respiratory syndrome coronavirus-2 (SARS-CoV-2), affected over 779 million people with approximately 7 million deaths worldwide since the outbreak in December 2019 [1]. The severity of SARS-CoV-2 infection varies widely among individuals, ranging from asymptomatic cases to critical disease that can lead to multiple organ failure and death. While most infected unvaccinated individuals have mild symptoms, 14% present more severe symptoms requiring hospitalization, and 5% become severely ill, requiring ventilation [2]. The host immune response plays a critical role in the pathogenesis of COVID-19, determining the clinical outcome of the disease, with dysregulation of host immunity being linked to the severity of the disease [3,4]. Following infection, the virus triggers the innate immune response, which includes the activation of macrophages and neutrophils, as well as the production of pro-inflammatory cytokines [5]. While that response generally aims to control viral replication, it can become dysregulated, leading to a cytokine storm. The adaptive immune response, including CD4^+^ T cells, CD8^+^ T cells, and B cells, is subsequently activated to target and eliminate the virus. However, in severe cases, that response may be inadequate or exacerbate inflammation, leading to critical disease [6].

The hyper-inflammatory state is characterized by the excessive release of cytokines such as interleukin-6 (IL-6), IL-2, IL-1β, granulocyte-colony stimulating factor (G-CSF), interferon gamma (IFN-γ), IFN-γ inducible protein-10 (IP-10), monocyte chemoattractant protein-1 (MCP-1), macrophage inflammatory protein (MIP)-1α, and tumor necrosis factor alpha (TNF-α), and it is considered to be the main cause of disease severity and death in patients with COVID-19 [7]. Indeed, patients admitted to the intensive care unit had higher concentrations of those cytokines than those with mild and moderate infections [8,9,10,11].

Likewise, lymphopenia has also been linked to disease progression. Key cytotoxic lymphocytic populations from innate and adaptive immune systems such as NK cells and CD8^+^ T cells are consistently reduced in COVID-19 patients, and they present immunity dysregulation such as functional exhaustion, and these changes have been associated with disease severity [5,12,13,14,15,16].

In the context of cytotoxic lymphocytes, gamma-delta (γδ) T cells play a critical and multifaceted role in antiviral immunity, as an interface between innate and adaptive immune responses. Although γδ T cells account for only 1–5% of circulating lymphocytes, they are highly present at barrier tissues such as the lung, gut, and skin, where they establish long-lived residency and acquire a pre-activated phenotype that enables rapid and non-redundant immune surveillance and effector responses upon infection [17].

The involvement of γδ T cells has been documented across multiple respiratory pathogens, including influenza viruses and SARS-CoV. Studies in seasonal influenza have shown that the Vγ9Vδ2 γδ T cell subset contributes to early antiviral defense by detecting virus-induced cellular stress and selectively targeting infected cells, resulting in effective control of viral replication [18]. Moreover, experimental evidence from highly pathogenic avian influenza H5N1 demonstrates a protective role for γδ T cells at the systemic level, as their absence is associated with increased mortality and exacerbated pulmonary pathology, and that H5N1 can directly activate human γδ T cells, inducing rapid cytokine production, including IFN-γ [19]. Consistent with these observations, evidence from SARS-CoV infection indicates that Vγ9Vδ2 γδ T cells undergo selective expansion following acute disease and exert antiviral activity through both cytotoxic mechanisms and IFN-γ-dependent non-cytolytic control of viral replication, supporting their role as multifunctional effectors in coronavirus infections [20]. In line with the aforementioned evidence, γδ T cells have not only been implicated in the immune response to SARS-CoV-2, but also have been associated with disease severity [21]. Jouan et al. (2020) showed no alteration in circulating γδ T cell relative proportions in severe COVID-19 patients, but other authors demonstrated a lower frequency of these cells in the blood of hospitalized COVID-19 patients when compared with healthy controls [22,23,24].

Lei et al. (2020) showed that γδ T cells from patients with mild symptoms of acute COVID-19 presented upregulation of the activation marker CD25, but no change in the exhaustion marker PD-1 [25]. However, activated HLA-DR^+^CD38^+^ and CD69^+^ (effector or terminally differentiated effector, TEMRA, profiles) and exhausted PD-1^+^ γδ T cells were increased in the circulation of acute COVID-19 patients with severe disease, compared with convalescent patients and/or uninfected individuals [21,22,26,27], suggesting the participation of these cells in the course of disease [21,22,26]. A gradual reduction is observed in Vδ2 T cells, but not in the Vδ1 subset, in acute COVID-19 patients, compared with COVID-19-recovered patients, and healthy ones. However, as the disease progresses, cell count for both Vδ1 and Vδ2 T decrease in peripheral blood, suggesting these cells infiltrate into the lungs [22,27].

Despite those preliminary data, how these cells could be implicated in SARS-CoV-2 response and/or immunopathogenesis of acute COVID-19 is still unclear. In the thymus, under the influence of innate programming and environmental signals, γδ T cells become functionally differentiated. An individual subset of γδ T cells possesses a restricted effector phenotype depending on the T cell receptor (TCR) expression, activation signal, and tissue distribution. However, depending on the signals received during disease or pathogen attack, even through innate immunity receptors, these committed cells can also be induced to show different effector properties, showing functional plasticity under ongoing immune response [28,29,30]. 

Considering that the functional plasticity of γδ T cells is driven by the inflammatory tissue microenvironment, and that the cytokine storm might modulate γδ T cell profile, it was hypothesized that acute COVID-19 patients with different disease severity upon SARS-CoV-2 infection present distinct intrinsic response potential of γδ T cells. Moreover, this study aimed to identify the intrinsic response potential of γδ T cells from COVID-19 patients with different illness severity, in order to better understand the implication of these cells in COVID-19 disease. For that purpose, γδ T cells from acute COVID-19 patients with moderate and severe diseases were isolated, stimulated in vitro, and evaluated for the expression of different functional molecules. Major changes were observed in the Vδ2 compartment in patients with more severe symptoms suggesting a more restricted response potential of γδ T cells in critical COVID-19. Furthermore, IL-17-producing Vδ2 cells were disrupted in more serious cases, also showing the impact of general immune dysregulation on these cells, or even suggesting some role for IL-17-producing Vδ2 cells in preventing critical symptoms.

## 2. Materials and Methods

### 2.1. Study Design and Population

This study is nested in the RECOVER-SUS study (NCT04807699), which is a prospective multicenter investigation including individuals hospitalized due to COVID-19 in seven public health centers in Brazil. It was conducted in accordance with the principles outlined in the Declaration of Helsinki (1975, revised in 2013) and was approved by the Research Ethics Committee from the “Instituto Nacional de Infectologia Evandro Chagas-Fundação Oswaldo Cruz” (INI/FIOCRUZ), Rio de Janeiro, Brazil (CAAE:32449420.4.1001.5262; Approval Code: 4.062.134). The RECOVER-SUS clinical cohort study, along with information on patient eligibility, enrollment, inclusion/exclusion criteria, and study design, has been previously described [31]. In the period of June to October 2020, about 302 unvaccinated in-patients from the cohort of the RECOVER-SUS study were admitted at INI/FIOCRUZ, and agreed to participate by written informed consent, of the immunological, virological and genetic investigative sub-study, from which the present study is included. All patients tested positive for SARS-CoV-2 by nasopharyngeal sampling using RT-qPCR. Heparinized blood samples were collected at the first 24 h from hospitalization and peripheral blood mononuclear cells (PBMC) were obtained by Ficoll-Paque density gradient centrifugation and cryopreserved until use. For this cross-sectional study, 44 participants were selected according to the clinical severity status and the availability and quality of biological samples for the investigation purpose (Figure S1).

### 2.2. Clinical Profiles

The study participants were selected in accordance with WHO Clinical Progression Scale [32] within the first 24 h of hospitalization, and classified in three groups based on disease severity: as moderate cases without oxygen support (MWO_2_; WHO:4; N = 15), moderate with non-invasive (nasal cannula or mask) oxygen support (MO_2_; WHO:5; N = 15), and severe disease (SD; WHO ≥ 7; N = 14), requiring mechanical ventilation support. Due to the lockdown and the inability to access patients who did not require hospitalization, mild acute COVID-19 cases could not be included in this study.

### 2.3. Ex Vivo Characterization of γδ T Cells

Total γδ T cells, and Vδ1 and Vδ2 subsets were characterized ex vivo from thawed PBMC. Almost 2 × 10^5^ cells were incubated for 20 min at room temperature in the dark, with antibodies to define T cell lineages: anti-CD3 (BV510, UCHT1 clone, BD Biosciences, San Jose, CA, USA), anti-TCRγδ (PE-CF594, B1 clone, BD Biosciences), anti-Vδ1 (PC7, R9.12 clone, Beckman-Coulter, Brea, CA, USA) and anti-Vδ2 (APC-Fire^TM^ 750, B6 clone, BioLegend, San Diego, CA, USA). Cells were washed and fixed with 2% paraformaldehyde prior to acquisition on equipment Cytoflex, from the Flow Cytometry Multiparametric Analysis Multiuser Facility, Instituto Oswaldo Cruz, Fundação Oswaldo Cruz, Rio de Janeiro, Brazil. Analyses were conducted using the Kaluza software version 2.3.0.20268 (Beckman-Coulter). T-distributed stochastic neighbor embedding (t-SNE) maps were generated from concatenated ex-vivo CD3^+^ events from 26 separate individuals under the same compensated parameters in the Cytobank platform (Beckman Coulter, CA, USA), using default software settings.

### 2.4. γδ T Cells Isolation

γδ T cells were purified by negative selection from thawed PBMC by magnetic beads using EasySep™ Human Gamma/Delta T Cell Isolation Kit (STEMCELL Technologies, Vancouver, BC, Canada), following the manufacturer’s instructions. The purity of γδ T cells was above 90%.

### 2.5. In Vitro γδ T Cell Intrinsic Response Potential Assay

The intrinsic response potential of γδ T cells was reached under in vitro stimulation with K562 myeloid lineage and OKT3 antibody. Isolated γδ T cells were cocultured overnight at 37 °C, with 5% CO_2_ at 1:1 ratio with the K562 cell lineage, as a cellular target, as previously described [33]. However, since the K562 cell line may not promote efficient activation of resting γδ T cells, OKT3 (Invitrogen, Thermo Fisher, Waltham, MA, USA) antibody was added to the culture as co-stimulus [30]. Stimuli with K562 and OKT3 alone were considered as baseline controls, since the low number of γδ T cells from the samples limited unstimulated control. Moreover, CD107a monoclonal antibody (FITC, H4A3 clone, BD Biosciences), brefeldin A (5 µg/mL; Sigma-Aldrich, St. Louis, MO, USA) and monensin (6 µg/mL; Sigma-Aldrich), were added concomitantly to the coculture. After 16 h of incubation, cells were stained with viability dye (Fixable Viability Stain 700, BD Biosciences), with a core panel of antibodies to define γδ T lineages (Table S1): anti-TCRγδ (PE-CF594, B1 clone, BD Biosciences), anti-Vδ1 (PC7, R9.12 clone, Beckman Coulter), and anti-Vδ2 (APC-Fire^TM^ 750, B6 clone, BioLegend), and with antibodies anti-CD27 (BV605, M-T271 clone, BD Biosciences) and anti-CD161 (PerCP-Cy5.5, HP-3G10 clone, BioLegend). In addition, the antibodies anti-TRAIL (BV421, RIK2 clone, BD Biosciences), anti-IFN-γ (PE, 4S.B3 clone, BD Biosciences), and anti-TNF-α (APC, MAb11 clone, BD Biosciences) defined panel 1, and the antibodies anti-IL-1R (PE, R&D Systems, Minneapolis, MN, USA), anti-IL-23R (APC, 218213 clone, R&D Systems), and anti-IL-17 (BV421, N49-653 clone, BD Biosciences) defined panel 2 (Table S1). Unstained and fluorescence minus one (FMO) were run in parallel as controls. Fixation and permeabilization were performed using IntraPrep Permeabilization Reagent (Beckman Coulter), following manufacturer instructions. The flow cytometry was performed on Cytoflex, and analyzed using Kaluza, as previously described. The flow cytometry gating strategy of analysis is presented in Figure S2. The frequency of positive cells and the median fluorescence intensity (MFI) of each parameter were considered for statistical analysis. MFI values were normalized by subtracting the corresponding unstained control. Some samples were not included in some analyses due to the low number of events. Finally, t-SNE maps were generated based on concatenated samples from MWO_2_ (N = 14), MO_2_ (N = 9), and SD (N = 9), using the Cytobank platform with default software parameters.

### 2.6. Statistical Analysis

Descriptive analyses of clinical and demographic characteristics were conducted using one-way analysis of variance (ANOVA) or the Kruskal–Wallis test for quantitative variables and Fisher’s exact test for categorical variables. For analyses of cell subsets and functional profiles, differences between the three clinical groups were reached with the Kruskal–Wallis test, followed by Post hoc multiple comparisons using Dunn’s test for independent samples. Alternatively, for variables presenting a tendency of increase/decrease in medians according to clinical severity and *p*-values < 0.1 at the Kruskal–Wallis test, the Jonckheere–Terpstra test was used to compare groups.

For paired analyses, evaluated groups were compared using the Friedman test, followed by the Wilcoxon matched-pairs signed-rank test. In addition, correlation analyses were performed using Pearson’s correlation coefficient. All analyses were performed using GraphPad Prism version 9.1.0 (GraphPad Software, San Diego, CA, USA) or RStudio software running R version 4.5.2 (R Foundation for Statistical Computing, Vienna, Austria). A *p*-value < 0.05 was considered statistically significant.

## 3. Results

### 3.1. Sociodemographic and Clinical Characteristics

The main sociodemographic and clinical data of all 44 individuals evaluated in the present study are described in Table 1. The median age observed was 63 years old (range 27–90), with 40.9% falling into the 60 to 80 age range. The gender distribution was balanced with 52% female and 48% male. The group included 28 (63.3%) brown, 9 (20.5%) white and 5 (11.4%) black skin color participants. Regarding previous comorbidities, systemic hypertension (36.4%) and diabetes (25.0%) were the most prevalent among the individuals. In hospital admission, symptoms such as dyspnea (72.2%), followed by cough (59.1%), oxygen saturation under 95% (43%), fever (43.2%) and myalgia (29.5%) were more frequently reported, while anosmia, ageusia, coryza (11.4%) and diarrhea (2.3%) were less reported. Patients were clustered into three groups based on COVID-19 severity and assessed for the parameters. The sequential organ failure assessment (SOFA) scores were consistently higher among SD patients [median: 8.5; 95% confidence interval (95% CI): 7.0–11.0] (*p* = 0.006), than among MWO_2_ (median: 1.0; 95% CI: 0–6.0) or MO_2_ (median: 3.0; 95% CI: 2.0–6.0) ones. These results were directly proportional to death (MWO_2_: 20.0%; MO_2_: 46.7%; SD: 87.5%) and indirectly proportional to discharge outcomes (MWO_2_: 80.0%; MO_2_: 46.7%; SD: 14.3%) (*p* = 0.002). Several laboratorial parameters were also investigated, and higher levels of creatinine were detected among SD patients (median: 1.53; 95% CI: 1.5–133.0) versus MWO_2_ (median: 1.05; 95% CI: 0.82–3.42) or MO_2_ (median: 0.98; 95% CI: 0.74–1.42) groups (Table S2).

### 3.2. Similar Frequencies of Vδ1 and Vδ2 Subsets Were Observed Among Moderate and Severe Patients

**Figure 1:**
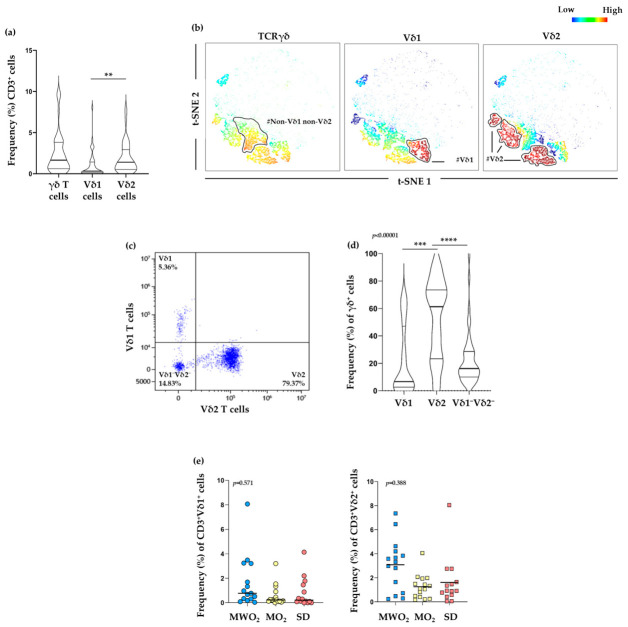
Ex vivo analyses of γδ T cells and Vδ1, Vδ2, and Vδ1^−^Vδ2^−^ subsets of moderate and severe COVID-19 in-patients. (**a**) Violin plots represent the frequency of circulating γδ T cells, Vδ1, and Vδ2 cells within CD3^+^ cells in the whole study group (N = 44). (**b**) t-SNE maps of γδ, Vδ1 and Vδ2 receptors expression based on CD3^+^ cells (N = 26). (**c**) Representative flow cytometry dot-plot of the Vδ1^+^, Vδ2^+^, and Vδ1^−^Vδ2^−^ subset frequency within CD3^+^γδ^+^ cells. (**d**) Violin plots of the frequency of Vδ1^+^, Vδ2^+^, and Vδ1^−^Vδ2^−^ subset frequency T cells within CD3^+^γδ^+^ in the whole study group (N = 44). (**e**) Dot-plots with CD3^+^Vδ1^+^ (left) and CD3^+^Vδ2^+^ (right) frequency distribution among MWO_2_ (N = 15), MO_2_ (N = 15) and SD (N = 14) patients. Medians and interquartile ranges are represented by horizontal bars in the graphs (**a**,**d**,**e**). Clinical study groups were analyzed by the Kruskal–Wallis test and *p*-values are indicated in the upper left corner of each graph. The Wilcoxon matched-pairs signed-rank test or Dunn’s test *p*-values < 0.05 were considered significant and are represented as: ** *p* < 0.01; *** *p* < 0.005; and **** *p* < 0.0005. MWO_2_ patients are represented in blue; MO_2_ patients are represented in yellow; SD patients are represented in red. Circles (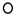) represent Vδ1^+^ cells; squares (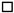) represent Vδ2^+^ cells.

Primarily, the frequency of circulating γδ T cells and their main Vδ1 and Vδ2 subsets were evaluated ex vivo, among total CD3^+^ T cells (Figure 1a), and a median frequency of 2.68% (95% CI: 0.95–2.37) was observed for γδ T cells, 0.32% (95% CI: 0.18–0.88) was observed for Vδ1 T cells, and 1.4% (95% CI: 0.89–1.99) for Vδ2 T cells. Consistently higher frequency of circulating Vδ2 T cells was noticed (*p* < 0.01). The relative expressions of Vδ1 and Vδ2 subsets within γδ T cells were assessed by performing t-SNE analysis (Figure 1b). While Vδ1 T cells appeared as a single cluster (cluster #1), Vδ2 T cells formed three distinct clusters (clusters #2–4). Interestingly, one more cluster (non-Vδ1 and non-Vδ2) was formed within γδ T cells (cluster #5), corresponding to Vδ1^−^Vδ2^−^ cells (Figure 1c). Within γδ T cells, a median frequency of 6.72% (95% CI: 4.29–24.49) was observed for Vδ1 T cells and a median frequency of 61.25% (95% CI: 36.74–70.46) for Vδ2 T cells, whereas 16.21% (95% CI: 13.64–26.32) was observed for Vδ1^−^Vδ2^−^ T cells (Figure 1d). The frequency of Vδ2 T cells was significantly higher than that of other subsets (*p* < 0.005 and *p* < 0.0005, respectively).

The frequency of γδ T cells, and Vδ1 and Vδ2 subsets among total CD3^+^ T cells did not differ between men and women (Figure S3). In the same way, no difference was observed among different age groups regarding the median frequencies of those cells (Figure S3). In addition, when these cells were analyzed in the milieu of COVID-19 severity, similar frequencies of circulating Vδ1 and Vδ2 cells were observed among MWO_2_ (Vδ1: 0.78%; 95% CI: 0.31–3.21; Vδ2: 3.32%; 95% CI: 0.73–4.2), MO_2_ (Vδ1: 0.23%; 95% CI: 0.13–0.91; Vδ2: 1.11%; 95% CI: 0.41–1.94), or SD patients (Vδ1: 0.21%; 95% CI: 0–1.78; Vδ2: 0.92%; 95% CI: 0.42–2.75) (Figure 1e). So, in the present study, the frequency of circulating Vδ1 and Vδ2 subsets did not change between moderate and critical disease.

### 3.3. Vδ1^−^Vδ2^−^ Subset Seems to Expand Under In Vitro Stimulation

To understand the role of γδ T cells in the pathogenesis of COVID-19, these cells were isolated from PBMC and exposed to in vitro overnight stimulation with both K562 target cells and αCD3-OKT3 and were assessed for the intrinsic response potential. Firstly, γδ T cells were assayed under K562 cells and OKT3 stimuli apart as controls, and low comparable frequencies were observed between them for every tried-out parameter (Figure S4). Conversely, each control alone triggered significantly less response of γδ T cells than stimulation with OKT3 in the presence of K562 cells, which induced higher cytokine production and cytotoxic degranulation, exceptionally for IL-17 production by OKT3 stimuli.

**Figure 2:**
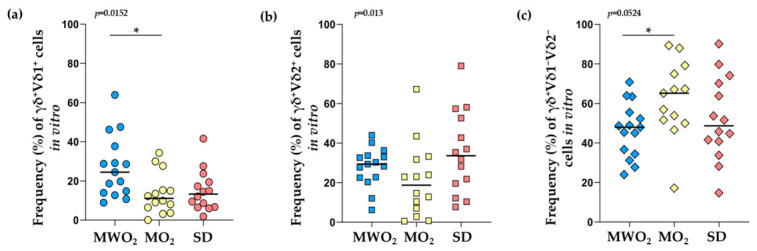
Frequency of Vδ1, Vδ2, and Vδ1^−^Vδ2^−^ subsets after in vitro stimulation. Dot-plots of frequency distributions of (**a**) γδ^+^Vδ1^+^, (**b**) γδ^+^Vδ2^+^, and (**c**) γδ^+^ Vδ1^−^Vδ2^−^ subsets among MWO_2_ (N = 15), MO_2_ (N = 14) and SD (N = 14) patients. Medians are represented by horizontal bars in the graphs. Clinical study groups were analyzed by the Kruskal–Wallis test and *p*-values are indicated in the upper left corner of each graph. Dunn’s test *p*-values < 0.05 were considered significant and are represented as: * *p* < 0.05. MWO_2_ patients are represented in blue; MO_2_ patients are represented in yellow; SD patients are represented in red. Circles (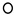) represent Vδ1^+^ cells; squares (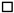) represent Vδ2^+^ cells; diamonds (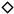) represent Vδ1^−^Vδ2^−^ cells.

After this initial analysis and following with in vitro stimulation, the frequencies of γδ T cell subsets were analyzed regarding COVID-19 severity. Patients from the MWO_2_ group (24.48%; 95% CI: 13.94–37.74) showed significantly higher median frequencies of the Vδ1 subset than MO_2_ patients (11.09%; 95% CI: 3.65–27.67) but not than SD ones (13.27%; 95% CI:6.49–23.72) (*p* < 0.05, Figure 2a). By contrast, no difference was observed among Vδ2 median frequencies from MWO_2_ (29.39%; 95% CI: 22.54–33.67), MO_2_ (18.74%; 95% CI: 2.87–33.27) and SD patients (33.61%; 95% CI: 12.20–57.41) (Figure 2b). In turn, a higher frequency of Vδ1^−^Vδ2^−^ cells was observed for MO_2_ patients (65.18%; 95% CI: 49.93–79.23) compared with MWO_2_ (47.93%; 95% CI: 34.4–55.46) (*p* < 0.05; Figure 2c), but not with SD ones (48.68%; 95% CI: 33.77–74.17). Interestingly, the proportion of Vδ1^−^Vδ2^−^ cells within γδ T cells seems to be increased after stimuli and encompassed in median up to 40% of total γδ T cells (Figure 2c). This observation may be noticed when set against the ex vivo profile (Figure S5), suggesting some in vitro expansion of these cells.

### 3.4. SD Patients Present γδ T Cells with Less Intrinsic Response Potential

**Figure 3:**
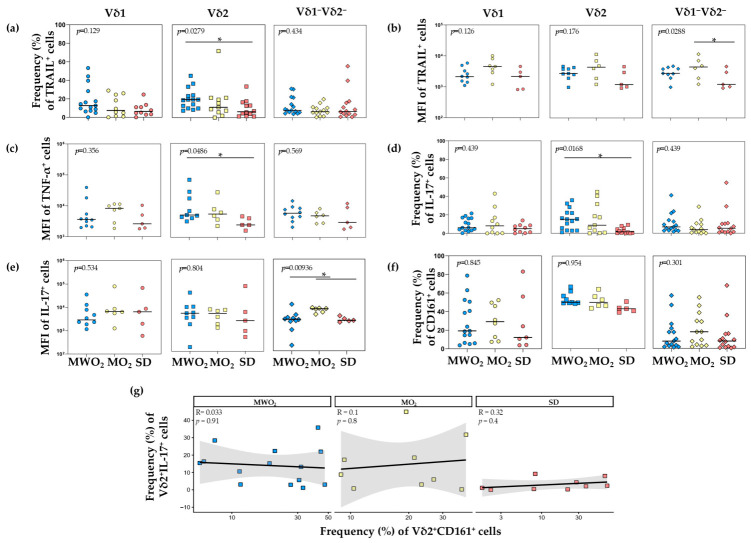
Analyses of functional markers in γδ T subsets after in vitro stimulation regarding the clinical presentation. (**a**) frequency of TRAIL-expressing cells, (**b**) MFI values of TRAIL expression, (**c**) MFI values of TNF-α expression, (**d**) frequency of IL-17-producing cells, (**e**) MFI values of IL-17 expression, (**f**) frequency of CD161-expressing cells, and (**g**) linear correlation between frequencies of CD161^+^ and IL-17-producing Vδ2 cells. Medians are represented by horizontal bars in the graphs. Clinical study groups were analyzed by the Kruskal–Wallis test and *p*-values are indicated in the upper left corner of each graph. Dunn’s test *p*-values < 0.05 were considered significant and are represented as: * *p* < 0.05. MWO_2_ patients are represented in blue; MO_2_ patients are represented in yellow; SD patients are represented in red. Circles (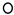) represent Vδ1^+^ cells; squares (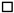) represent Vδ2^+^ cells; diamonds (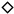) represent Vδ1^−^Vδ2^−^ cells. In linear correlation, R and *p*-values are indicated in the graphs.

To characterize the intrinsic response potential from γδ T cells under in vitro stimulation, these cells were assessed for the expression of some functional markers. Since γδ T cells can utilize death receptor ligands, such as TRAIL, to mediate cytotoxicity against virus-infected cells, that molecule was targeted. Higher frequency of Vδ2 cells expressing TRAIL, upon stimuli, was consistently observed in MWO_2_ patients (19.10%; 95% CI: 9.91–33.33) than SD patients (5.82%; 95% CI: 1.34–16.52), but not than MO_2_ patients (11.14%; 95% IC: 5.83–21.40) (*p* < 0.05; Figure 3a).

In parallel, the analysis of the normalized MFI showed that the expression of TRAIL on Vδ1^−^Vδ2^−^ cells was significantly higher for MO_2_ (3924; 95% CI: 1623–7558) compared with SD patients (1479; 95% CI: 896.6–2163) (*p* < 0.05), whereas no difference was observed for Vδ1 or Vδ2 among the clinic groups (Figure 3b). Similarly, significantly higher values of MFI_TNF-α_ were observed in Vδ2 cells, but not in Vδ1 or Vδ1^−^Vδ2^−^ cells, in MWO_2_ patients (5974; 95% CI: 3196–9474) compared with SD patients (3020; 95% CI: 1834–12,188) (*p* < 0.05; Figure 3c). In turn, comparable frequencies of Vδ1, Vδ2 and Vδ1^−^Vδ2^−^ subsets expressing TNF-α, CD107a, MIF_CD107a_, and IFN-γ, or MIF_IFN-γ_ values were observed among the patients regardless of disease severity (Figure S6).

Considering γδ T cells can be an innate source of IL-17 and therefore be polarized toward IL-17 production under specific conditions [34], these cells were also evaluated for intracellular expression of IL-17. The analysis in the context of disease severity showed a consistently higher frequency of Vδ2 cells expressing intracellular IL-17, but not of Vδ1 or Vδ1^−^Vδ2^−^ cells, in MWO_2_ patients (14.26%; 95% CI: 3.02–22.37) compared with SD patients (2.07%; 95% CI: 0.29–7.98) (*p* = 0.0172; Figure 3d). Of interest, a trend toward a decrease in IL-17 frequency with disease severity in Vδ2 cells was also observed, which was significant in the Jonckheere–Terpstra tendency test (*p* = 0.0058). However, the comparison of MFI distributions detected for the IL-17-producing γδ T subsets revealed differences only to Vδ1^−^Vδ2^−^ cells, with higher values being observed for MO_2_ patients (6839; 95% CI: 3975–7650) compared with MWO_2_ (2393; 95% CI: 842.5–3784) or SD ones (2142; 95% CI: 1787–3414) (*p* < 0.05; Figure 3e).

Thus, the distinctive marker of IL-17-producing cells [35]―the human C-type lectin-like molecule, CD161―was also investigated, as well as the receptors to the critical cytokines for human γδ T cell IL-17 polarization: IL-1β and IL-23 [34]. For the Vδ2 subset, it was expected that the frequency of CD161^+^ cells would follow the same profile observed in IL-17-producing cells (Figure 3d), and also that a lower frequency of CD161-expressing cells would be observed in the SD patients, in relation to the MWO_2_ ones. Nevertheless, no difference was observed in the frequency of CD161^+^ Vδ2 cells, nor in Vδ1, nor in Vδ1^−^Vδ2^−^ subsets, among the clinically distinct patients (Figure 3f). No significant linear correlation was observed between CD161^+^ and IL-17-producing Vδ2 cell frequencies, regardless of clinical presentation (Figure 3g).

**Figure 4:**
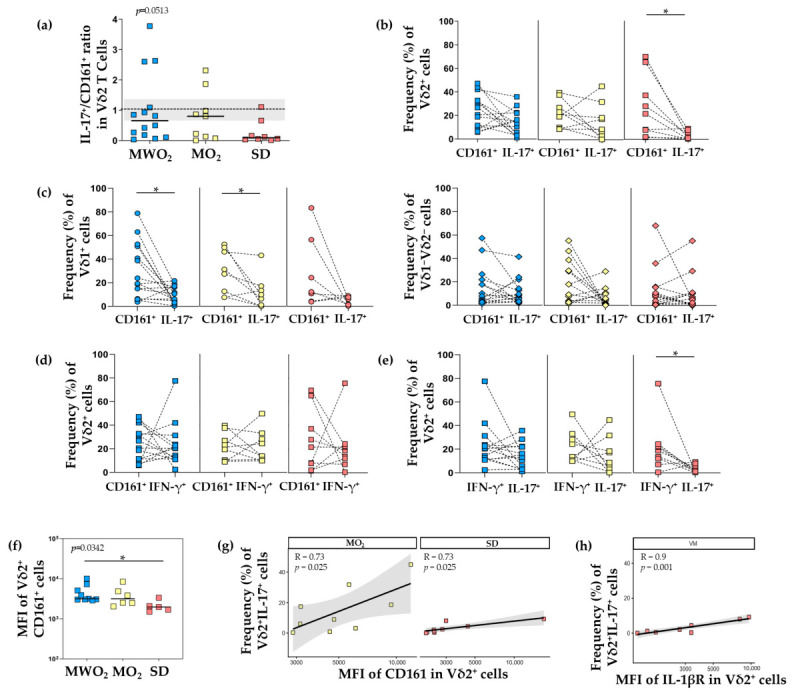
IL-17, CD161, IFN-γ, and IL-1βR expression patterns and correlations in γδ T cell subsets after in vitro stimulation regarding the clinical presentation. (**a**) Ratio of frequency of IL-17-producing and CD161^+^ Vδ2 cells, (**b**) paired analysis between frequencies of CD161^+^ and IL-17-producing Vδ2 cells, (**c**) paired analysis between frequency of CD161^+^ and IL-17-producing Vδ1, and Vδ1^−^Vδ2^−^ subsets, (**d**) paired analysis between frequencies of CD161^+^ and IFN-γ-producing Vδ2 cells, (**e**) paired analysis between frequencies of IFN-γ- and IL-17-producing Vδ2 cells, (**f**), MFI values of CD161 expression in Vδ2 cells, (**g**) linear correlation between frequencies of IL-17-producing Vδ2 cells and MIF values of CD161 expression of MO_2_ and SD patients, and (**h**) linear correlation between frequencies of IL-17-producing Vδ2 cells and MIF values of IL-1βR expression in SD patients. Medians are represented by horizontal bars in the graphs. Clinical study groups were analyzed by the Kruskal–Wallis test and *p*-values are indicated in the upper left corner of each graph. The Wilcoxon matched-pairs signed-rank Test or Dunn’s test *p*-values < 0.05 were considered significant and are represented as: * *p* < 0.05. MWO_2_ patients are represented in blue; MO_2_ patients are represented in yellow; SD patients are represented in red. Circles (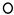) represent Vδ1^+^ cells; squares (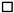) represent Vδ2^+^ cells; diamonds (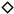) represent Vδ1^−^Vδ2^−^ cells.

To further this analysis, the ratio of IL-17 and CD161 frequencies and the paired expression of both molecules were assessed. Interestingly, for Vδ2 cells, most of the patients from the three clinical groups presented the IL-17/CD161 ratio < 1.0 (MWO_2_: 71%; MO_2_: 67%; SD: 87.5%), while only some of them presented values around 1.0 (>0.75<1.25) (MWO_2_: 29%; MO_2_: 33%; SD: 12.5%), considering the ratio of one-to-one for the frequency of cells expressing these molecules (Figure 4a). Statistical analysis revealed for Vδ2 cells a near-significant difference among the groups (*p* = 0.0513), as well as a near-significant post hoc pairwise comparison between the MWO_2_ and SD patients (*p* = 0.054).

In turn, paired analysis for the frequencies of CD161^+^ and IL-17-producing Vδ2 cells in patients from each different clinical study group allowed us to observe this relationship more clearly. Thus, significantly lower frequency of IL-17^+^ than CD161^+^ Vδ2 cells was observed in SD patients, but not in MWO_2_ or MO_2_ patients (*p* = 0.012; Figure 4b). For Vδ1 cells, the low proportion of IL17-producing cells in relation to those CD161^+^ cells was more pronounced and reached significance in MWO_2_ and MO_2_ patients (*p* < 0.05), while for the Vδ1^−^Vδ2^−^ subset no significant difference was observed (Figure 4c). Taken together, these observations revealed a disproportion on frequency between CD161^+^ and IL-17^+^ γδ T cells among all the patients, which was even more pronounced in SD patients for the Vδ2 subset, due to the consistent reduction in IL-17-producing cells in these individuals.

Conventional CD161^+^ CD4^+^ T cells can also produce IFN-γ, even simultaneously with IL-17, under certain circumstances [36,37,38]. Considering that this could also take place within γδ T cells, paired analysis for the frequencies of CD161^+^ and IFN-γ^+^ Vδ2 cells was done. The frequency of CD161^+^ Vδ2 cells matched the frequency of IFN-γ-producing ones in all patients, and no difference was observed among the clinical groups (Figure 4d). Nevertheless, when the frequencies of IFN-γ- and IL-17-producing Vδ2 cells were paired, significantly fewer IL-17^+^ cells than IFN-γ^+^ cells were observed in SD patients (*p* < 0.05; Figure 4e), reinforcing the idea that these patients in a more severe condition had Vδ2 cells with a reduced ability to produce IL-17.

Lastly, analysis of MIF_CD161_ values for γδ T showed that Vδ2 cells expressed significantly lower values in SD (1972; 95% CI: 1510–3350) than in MWO_2_ patients (3163; 95% CI: 3015–7304) (*p* < 0.05; Figure 4f). No difference was observed in MIF_CD161_ values expressed by Vδ1 or Vδ1^−^Vδ2^−^ cells among patients (Figure S7). Interestingly, in the Vδ2 subset, a significant linear correlation was observed between the frequency of IL-17^+^ cells and the values of MIF_CD161_ in MO_2_ (R = 0.73; *p* = 0.025) and in SD (R = 0.73; *p* = 0.025) patients, implying that CD161 expression level might have influenced the frequency of IL-17-producing Vδ2 cells in these patients (Figure 4g).

The receptors to the critical cytokines involved in human γδ T cell IL-17 polarization [34], IL-1β and IL-23, were also investigated. However, neither the frequency of Vδ1, Vδ2, and Vδ1^−^Vδ2^−^ cells expressing these molecules nor the MIF values for these receptors differ among patients (Figure S7). In turn, significant linear correlation was observed for the Vδ2 subset considering the frequency of IL-17^+^ cells, and MFI_IL-1βR_ in SD patients (R = 0.9; *p* = 0.001; Figure 4h). As observed in MIF_CD161_ values, the frequency of IL-17-producing Vδ2 cells in these patients might have been influenced by the levels of expression of IL-1β receptor.

**Figure 5:**
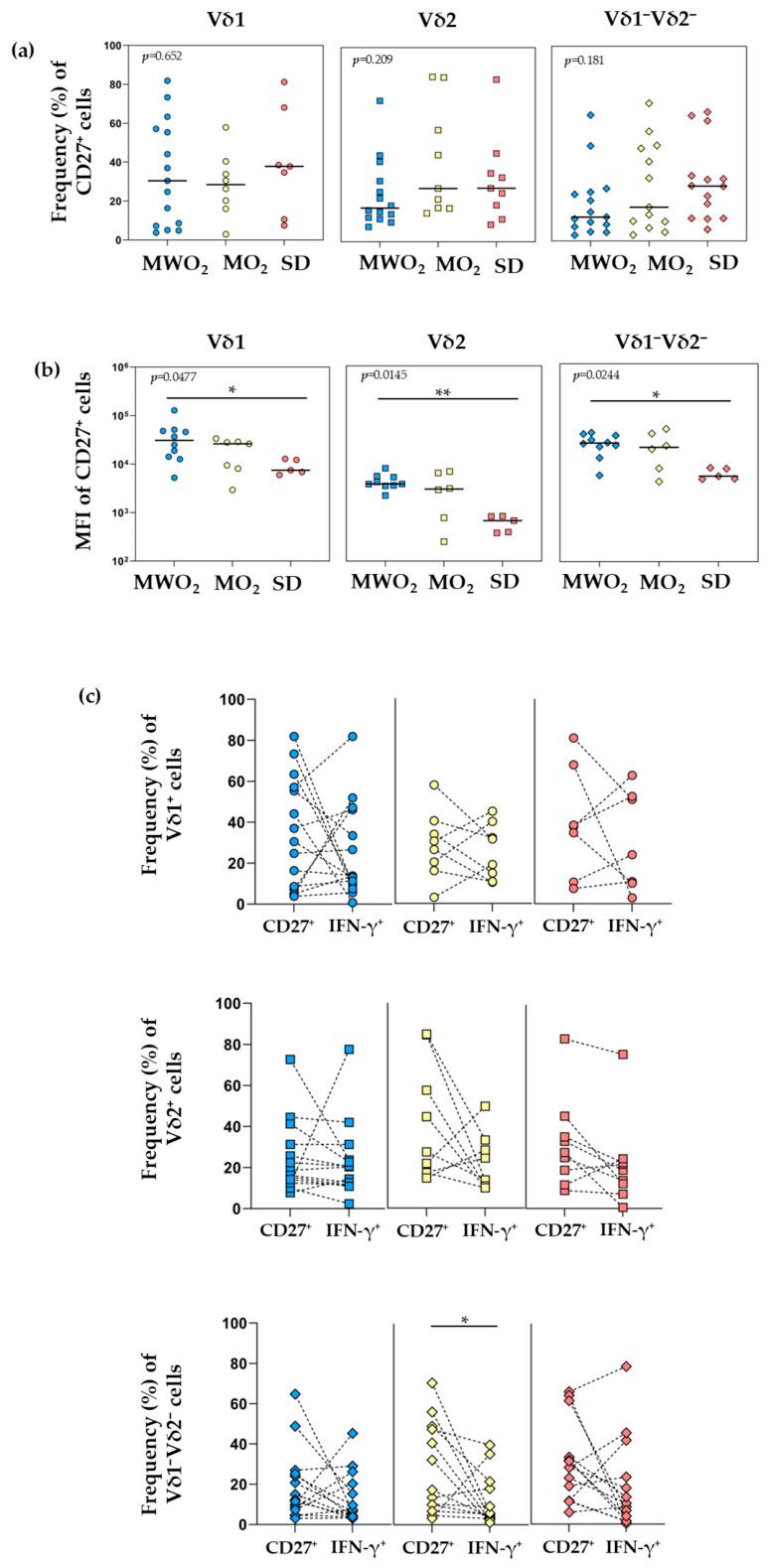
CD27 expression patterns and correlations with IFN-γ in γδ T cell subsets after in vitro stimulation regarding the clinical presentation. (**a**) Frequency of CD27^+^ cells, (**b**) MFI values of CD27 expression, and (**c**) paired analyses between frequency of CD27^+^ and IFN-γ-producing Vδ1, Vδ2, and Vδ1^−^Vδ2^−^ subsets. Medians are represented by horizontal bars in the graphs. Clinical study groups were analyzed by the Kruskal–Wallis test and *p*-values are indicated in the upper left corner of each graph. The Wilcoxon matched-pairs signed-rank test or Dunn’s test *p*-values < 0.05 were considered significant and are represented as: * *p* < 0.05; ** *p* < 0.01. MWO_2_ patients are represented in blue; MO_2_ patients are represented in yellow; SD patients are represented in red. Circles (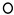) represent Vδ1^+^ cells; squares (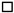) represent Vδ2^+^ cells; diamonds (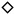) represent Vδ1^−^Vδ2^−^ cells.

Once the tumor necrosis factor receptor family member CD27 can act as a coreceptor for γδ T cells activation and even participates in the functional differentiation of these cells, it was also investigated. In general, the frequency of CD27^+^ Vδ1, Vδ2, and Vδ1^−^Vδ2^−^ cells did not differ among the patients with different clinical presentations (Figure 5a). Interestingly, the median frequency of CD27^+^ cells did not achieve values greater than 40% for all γδ T subsets in any of the groups. Not only that, but significant lower MIF_CD27_ values were observed for Vδ1, Vδ2, and Vδ1^−^Vδ2^−^ cells in SD patients (CD27_Vδ1_: 7490, 95% IC: 5987–12,867; CD27_Vδ2_: 4202, 95% IC: 2722–4922; CD27_Vδ1−Vδ2−_: 5350, 95% IC: 4661–7901, respectively) when compared with MWO_2_ patients (CD27_Vδ1_: 30,846, 95% IC: 12,628–51,527; CD27_Vδ2_: 15,324, 95% IC: 14,326–20,327; CD27_Vδ1−Vδ2−_: 25,027, 95% IC: 12,600–38,370, respectively) (*p* < 0.05; Figure 5b). Paired analysis for the frequencies of CD27^+^ and IFN-producing cells showed no difference between these molecule frequencies, except for the higher frequency of CD27 among Vδ1^−^Vδ2^−^ cells in the MO_2_ patients (*p* < 0.05), revealing, in a general way, a corresponding proportion of Vδ1, Vδ2, and Vδ1^−^Vδ2^−^ CD27^+^ and IFN-producing cells in the patients, regardless of the clinical presentation (Figure 5c). Therefore, for CD27^+^ γδ T subsets, a close correspondence with IFN-producing cells was observed, implying these cells could be involved in the same pathway.

**Figure 6:**
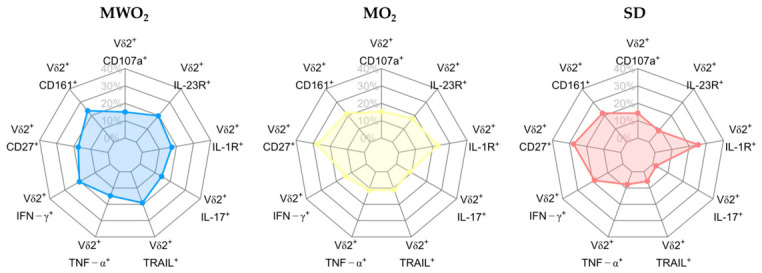
Cytokine and surface markers expression patterns in Vδ2^+^ cells across clinical groups revealed by radar plots. The MWO_2_ group is represented in blue. The MO_2_ group is represented in yellow. SD group is represented in red.

When the parameters were taken into account together, the radar plot analysis highlighted distinct functional programming of Vδ2^+^ T cells across clinical groups (Figure 6). Notably, IL-17 detection was relatively higher in the patients compared with the more severe ones, suggesting that IL-17 production by Vδ2^+^ T cells is more preserved and might be associated with a more effective or regulated immune response in milder disease. In contrast, SD patients exhibited reduced IL-17-producing Vδ2^+^ T cells although some classic markers associated with the Th17 profile were preserved in these patients, suggesting some potential functional impairment or exhaustion of these cells.

In complement and considering the generated dataset, t-SNE maps were constructed to visualize the global phenotypic distribution of Vδ2 T cells across the three clinical groups of COVID-19 severity (Figure S8). Since the study employed two antibody panels, two t-SNE signatures were observed, each one capturing a different facet of the cellular phenotype. Across both panels, the maps showed a progressive reduction in structural definition with increasing disease severity, accompanied by heterogeneity in marker expression, as revealed by radar plots (Figure 6).

### 3.5. Patients with Favorable Outcome Present Cytotoxic Behavior

Considering the two clinical outcomes observed in this study, it was hypothesized that γδ T subsets with different intrinsic response potential would be observed. The patients were classified as a favorable outcome in case of hospital discharge, or unfavorable outcome in cases of death.

Firstly, the ex vivo frequency of circulating γδ T, Vδ1, and Vδ2 subsets was evaluated among total CD3^+^ T cells. Patients with unfavorable outcome showed lower median frequency of circulating γδ T, Vδ1, and Vδ2 subsets (γδ: 0.91%, 95% CI: 0.46–1.71; Vδ1: 0.26%, CI: 0.11–0.88; Vδ2: 0.83%, CI: 0.41–1.47) than those with favorable outcome (γδ: 3.26%, 95% CI: 1.15–4.22; Vδ1: 0.84%, CI: 0.26–3.2 Vδ2: 2.89%, CI: 1.33–4.06) (*p* < 0.005, *p* < 0.05, *p* < 0.0005, respectively). No difference was observed for ex vivo or in vitro analysis of frequency of γδ^+^Vδ1^+^, γδ^+^Vδ2^+^, and γδ^+^Vδ1^−^Vδ2^−^ subsets (Figure S9).

**Figure 7:**
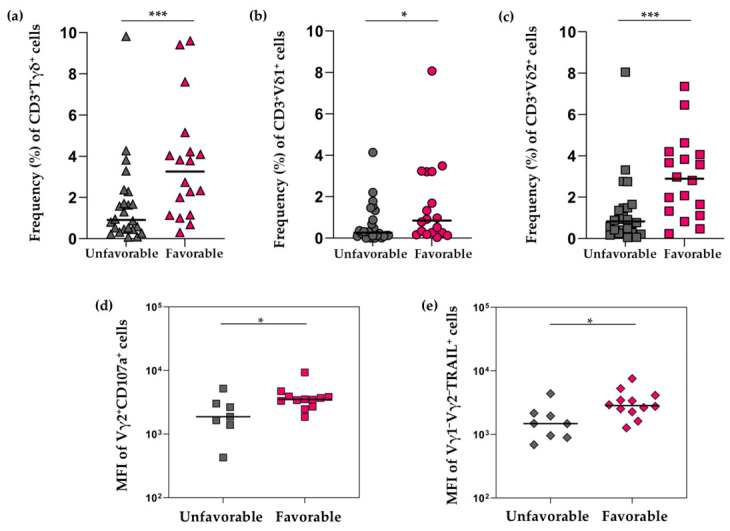
Analyses ex vivo and after in vitro stimulation of γδ T cells and Vδ1, Vδ2, and Vδ1^−^Vδ2^−^ subsets regarding the clinical outcomes. Dot-plots show (**a**) CD3^+^γδ^+^, (**b**) CD3^+^Vδ1^+^, and (**c**) CD3^+^Vδ2^+^ frequencies ex vivo, and (**d**) MFI values of CD107a in Vδ2 cells, and (**e**) MFI values of TRAIL in Vδ1^−^Vδ2^−^ cells, comparing patients with unfavorable (death) and favorable (hospital discharge) outcomes. Medians are represented by horizontal bars in the graphs. The Wilcoxon matched-pairs signed-rank test *p*-values < 0.05 were considered significant and are represented as: * *p* < 0.05; and *** *p* < 0.005. An unfavorable outcome is represented in gray. A favorable outcome is represented in pink. Triangles (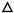) represent total CD3^+^γδ^+^ cells, circles (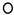) represent Vδ1^+^ cells; squares (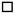) represent Vδ2^+^ cells; diamonds (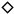) represent Vδ1^−^Vδ2^−^ cells.

Moreover, and unexpectedly, no significant difference was observed in the different parameters analyzed between favorable and unfavorable outcomes, nor in Vδ1, nor in Vδ2, or in Vδ1^−^Vδ2^−^ cells. Exceptions were observed in the MIF_CD107a_ values, higher in the Vδ2 cells, and to the MIF_TRAIL_ values, consistently higher in the Vδ1^−^Vδ2^−^ cells, among patients with favorable outcome (Vδ2-MIF_CD107a_: 3503; 95% CI: 2697–3920, Vδ1^−^Vδ2^−^-MIF_TRAIL_: 2851; 95% CI: 2276–4131), comparing with those with unfavorable outcome (Vδ2-MIF_CD107a_: 1873; 95% CI: 429–5206, Vδ1^−^Vδ2^−^-MIF_TRAIL_: 1486; 95% CI: 691.3–4383) (*p* = 0.03 and *p* = 0.01; Figure 7). These results imply that patients with favorable outcomes preserved circulating γδ T cells in the periphery and that the Vδ2 subset might have a greater cytotoxic potential under stimulation.

## 4. Discussion

This study provided an overview of the intrinsic response potential of γδ T cells in acute COVID-19 in patients with moderate symptoms and more critical disease. The results showed that hospitalized patients with severe disease who required mechanical ventilation presented Vδ2 cells with a more compromised functional profile.

In a group of 44 acute COVID-19 patients ranging from moderate to severe symptoms, the risk factors for serious disease such as advanced age, diabetes, and hypertension were likewise observed among the cases [39]. Moreover, the balanced distribution of patients across different age groups and genders within the clinical groups minimizes potential bias associated with these variables on the immune response [40]. Clinical stratification was supported by progressively higher SOFA scores and mortality rates in the severe group, supporting the biological distinction between patient categories [41].

Ex vivo analysis of circulating γδ T cell frequency revealed a predominance of the Vδ2 subset, among CD3^+^ T lymphocytes and within the γδ T compartment. This distribution mirrors the classical γδ T cell profile in healthy adult individuals [42], indicating that, despite the inflammatory context associated with COVID-19, the basal architecture of the γδ T compartment remains at least partially preserved in the peripheral circulation, in contrast to other viral infections [22,43,44,45]. Interestingly, t-SNE analysis revealed some heterogeneity among Vδ2 cells, which was organized into distinct clusters, whereas Vδ1 cells formed a more homogeneous grouping. Consistent with previous reports describing the functional plasticity and activation-driven phenotypic shifts in Vδ2 cells during SARS-CoV-2 infection, this diversity may reflect different states of activation, differentiation, or responses to the systemic inflammatory microenvironment [46,47]. In addition, alterations concerning CD3 expression on γδ T cells have also been observed under chronic inflammatory conditions [48]. In the context of SARS-CoV-2 infection, γδ T cells and particularly Vδ2 subsets exhibit altered phenotypes and activation markers, consistent with diverse functional states induced by systemic inflammatory signals [25,46].

Moreover, the identification of a substantial fraction of non-Vδ1 non-Vδ2 cells, which may be related to the Vδ3^+^ subset, highlights the complexity of the γδ T cell compartment implication during SARS-CoV-2 infection. Previous study has reported the presence and relative expansion of this uncommon γδ T cell subset in the context of severe COVID-19 [22], suggesting that its accumulation in peripheral blood reflects a broader inflammatory milieu and a global activation state rather than a uniform response across γδ T subpopulations. Clonal expansion of Vδ3 cells has also been observed in CMV and hepatitis C virus infections and has been associated with viral control [49,50], highlighting an important role for these cells in viral diseases. This third γδ T subset is rare in blood, composing around 0.2% of circulating T cells, but is enriched in liver and gut epithelium [48,51]. Therefore, in the context of COVID-19, this γδ T cell subset should be further investigated especially considering that SARS-CoV-2 also infects enterocytes and causes dysbiosis [52,53,54].

Quantitative changes in circulating γδ T cell frequencies have been reported in patients with COVID-19 and were associated with disease severity. Lei et al. (2020) demonstrated a robust reduction in peripheral γδ T cells in patients with severe disease compared with mild clinical presentations [25]. In the present study no reduction in peripheral γδ T cells was observed among moderate and severe COVID-19 cases, showing that the cell frequencies were impacted in a comparable way in these patients, despite the clinical differences.

The increased expression of markers associated with cytotoxicity and functional activation in the γδ T compartment from patients with severe disease has been suggested as a potential contribution of these cells to SARS-CoV-2-infection-associated tissue damage. In the context of severe COVID-19, heightened γδ T cell activation has been associated with increased production of proinflammatory mediators, supporting a role for these cells in amplifying local and systemic inflammatory responses [25,46]. To investigate the intrinsic response potential of γδ T cells across COVID-19 severity, these cells were exposed to in vitro stimuli. A key limitation in the field is the deficit of knowledge about γδ T cell targets. Although several known molecules activate these cells via TCR, many others can induce their responses through diverse innate and adaptive pathways [55]. In order to reach an overall activation without interfering with its intrinsic response potential, the classical NK assay using K562 lineage was adapted to γδ T cells. Stimulation with K562 cells or with the αCD3 antibody OKT3 alone resulted in similarly low responses. In contrast, combined stimulation with OKT3 and K562 cells promoted a significant increase in cytokine production and cytotoxic degranulation, validating the experimental model as an effective system to assess γδ T cell function. As previously shown, K562 cells can interact with isolated γδ T cells via TCR, but efficient responses require prior activation, which was achieved in the presence of OKT3 [56]. In turn, OKT3 alone and along with K562 induced similar frequencies of IL-17-producing γδ T cells, suggesting a limited potential of IL-17 production.

Following in vitro stimulation, hospitalized patients with moderate COVID-19 who did not require oxygen therapy exhibited a significantly higher frequency of Vδ1 cells, suggesting some preserved proliferative capacity of this subset. Interestingly, a proportional increase in Vδ1^−^Vδ2^−^ cells took place within the total γδ T cells, which may reflect in vitro expansion of this subset. This third γδ T subset was also observed ex vivo, and its expansion in vitro has occurred regardless of the clinical group, showing an overall preserved potential of proliferation. Considering these cells as being the Vδ3 subset, they have cytotoxic potential and the ability to produce Th1, Th2 and Th-17 cytokines [57], indicating functional plasticity similar to other γδ T subsets, and may be engaged in the response against SARS-CoV-2.

Some functional markers were assessed in γδ T cells after in vitro stimulation and major changes were predominantly observed in the Vδ2 compartment, which were consistent with disease severity. Analysis of cytotoxic related molecules, such as TRAIL and TNF-α, revealed a gradual functional impairment of γδ T subsets, from the less to the more severe patients. Despite only _MIF_TNF-α, but not the frequency of TNF-α^+^ cells, being reduced, some impairment can also be considered. Previous studies have shown a significant reduction in these molecules in COVID-19 patients, encompassing other cells, such as NK cells, and even presented the reduction in TRAIL as a predictor of progression of SARS-CoV-2 infection [58,59]. This decrease may reflect cellular exhaustion associated with persistent systemic inflammation [46], or even loss of function under extreme conditions. On the other hand, the preservation of these molecules in moderate cases may be essential to support functional response of the cytotoxic lymphocytes, including γδ T cells, against the virus, and help to avoid disease progression.

γδ T cells are well-established innate sources of IL-17, especially in mucosal tissues [60], and can also be polarized toward IL-17 production under specific conditions [34]. In this context, the IL-17 was targeted here, and the results have raised some points, especially regarding the Vδ2 cells. Previous study showed that severe COVID-19 patients presented a higher frequency of IL-17-producing γδ T cells than healthy donors under in vitro pan-stimulation with phorbol 12-myristate 13-acetate and ionomycin [22]. In the same way, Di Simone et al. (2022) reported a higher frequency of IL-17-producing Vδ2 cells in moderate acute COVID-19 patients than controls [61]. However, that difference was missed under stimulation of TCR with zoledronate, implicating that the activation pathway impacts the results. In the present study, patients with moderate COVID-19 exhibited frequency of IL-17-producing cells comparable to those stimulated with zoledronate [61]. However, patients with severe disease lacked IL-17-producing Vδ2 cells. Since these patients were classified on the basis of oxygen demanding, it was expected that more clinically severe patients requiring mechanical ventilation support should present higher frequency of IL-17-producing cells, because hypoxia has been associated with IL-17 profile and tissue repair [62,63]. Therefore, the results are suggestive of a more compromised functional profile presented in these patients with more severe symptoms.

Although different studies have implicated the IL-17-driven responses in COVID-19-associated immunopathology [64], the results here observed suggest a different scenario in which IL-17-producing Vδ2 cells could have some positive role and would not be associated with less critical stages of infection. In mice, neonatal influenza intranasal infection resulted in accumulation of IL-17-producing γδ T cells in the lungs, which was critical to protect animals from lung pathology and to increase survival rates [65]. The mechanism behind that was the production of IL-33 by lung epithelia cells, induced by IL-17, which was in turn responsible for the recruitment of T regulatory cells, and the induction of amphiregulin, a factor that promotes tissue repair [65,66]. Such IL-17–IL-33–amphiregulin association was also observed in nasal washes of human infants with influenza infection and presenting mild symptoms [65].

Following these findings and contrary to expectations, CD161^+^ cell frequencies did not linearly mirror IL-17 production across clinical groups. In particular, within the Vδ2 subset, the lack of a direct association between CD161 expression and IL-17 output suggests that CD161 alone may not reliably predict IL-17-producing capacity in the context of SARS-CoV-2 infection, where the inflammatory milieu may uncouple phenotypic markers from effector function. This finding contrasts with previous reports describing CD161 as a hallmark of IL-17-competent T cell populations, including γδ T cells, under non-COVID settings [35,67].

Analyses of the association between IL-17 and CD161 showed a more pronounced imbalance in patients with severe disease, characterized by the preservation of CD161^+^ Vδ2 cells alongside a reduction in the fraction effectively producing IL-17. This dissociation was even more pronounced among Vδ1. Rather than indicating a global loss of Vδ2 activity, this pattern suggests a selective disruption of IL-17 effector programming under COVID-19 severe inflammatory conditions. In parallel, the maintained correspondence between CD161^+^ and IFN-γ^+^ cells supports the notion that Vδ2 functional capacity is not abolished in severe disease, but may be qualitatively reprogrammed, potentially favoring Th1-associated outputs over IL-17 responses in this group.

Furthermore, the positive correlation between the levels of expression of IL-1βR and the frequency of IL-17-producing Vδ2 cells suggests that responsiveness to IL-1β-related inflammatory cues is at least partially preserved in those cells, in line with the established role of IL-1β-dependent signaling in promoting IL-17 competence in γδ T cells [35,68]. However, the overall reduction in these expression levels in severe patients may constrain efficient polarization toward IL-17 despite the persistence of CD161^+^ cells.

Finally, although the frequencies of CD27^+^ cells did not differ among clinical groups, the consistent reduction in CD27 expression intensity across γδ T cell subpopulations in severe disease suggests impaired costimulatory signaling quality. Given the association of CD27 with γδ T cell activation state and IFN-γ-biased effector potential, reduced CD27 signaling strength could contribute to suboptimal activation efficiency even in the presence of responsive cells [67,69].

Collectively, these findings raise the idea that severe COVID-19 can be associated with qualitative alterations in the γδ T cell compartment functional reprogramming in the γδ T cell compartment characterized by reduced TRAIL-mediated cytotoxicity, selective loss of IL-17 and partial preservation of IFN-γ production, and reduced costimulatory signaling, especially regarding Vδ2 cells. This functional dysregulation might contribute to more ineffective immune responses and to the worsening of clinical presentation. In addition, the relatively preserved IL-17 compartment of Vδ2 cells might have some positive role in less severe cases, such as tissue repair.

Given the divergent clinical outcomes observed in this cohort, it was hypothesized that γδ T cell subsets with distinct intrinsic response potentials would also be associated with favorable or unfavorable outcomes. However, the absence of significant differences in the ex vivo frequencies of circulating γδ T cells and their major subsets between discharged and deceased patients indicates that quantitative alterations alone are insufficient to discriminate clinical outcome in COVID-19.

Unexpectedly, most of the evaluated functional parameters under in vitro stimulation also failed to distinguish between clinical outcomes across γδ T cell subsets. Perhaps the cross-sectional design of analyses limited to hospitalization had restricted the scope of observation and resulted in the loss of important information that could have allowed the association between the observed disorders, disease severity, and clinical favorable or unfavorable outcomes.

Notably, patients with favorable outcomes displayed significantly higher expression of cytotoxicity-associated markers―namely CD107a―in Vδ2 cells, and TRAIL in Vδ1^−^Vδ2^−^ cells. The association between enhanced degranulation capacity and death receptor-mediated cytotoxicity with favorable clinical outcomes suggests that effective γδ T cell-mediated cytotoxic responses may contribute to viral control while limiting excessive immune activation. In contrast, reduced cytotoxic potential in patients with unfavorable outcomes may reflect functional exhaustion or impaired effector signaling, potentially contributing to uncontrolled viral replication or prolonged inflammation.

This study has some limitations that should be considered. The main limitation was the relatively small sample size, encompassing three clinical groups. Although a substantial number of participants were recruited for the sub-study in which the present study is nested, the patients were assigned to reach other different hypotheses from our group, which limited the sample size evaluated here. Furthermore, the general lymphopenia associated with COVID-19 limited the PBMC recovery and the subsequent cell availability for the assays, and consequently, the number of samples that could be included across the assessments. Because of the sample size, multivariate analyses were not carried out, which restricted the statistical analysis to univariate analyses and consequently, the statistical power of the analyses. To mitigate these limitations, appropriate non-parametric statistical approaches were applied, and rigorous flow cytometry controls, including FMO and negative controls, were used to ensure data reliability. Moreover, comparisons were carefully performed within clinically defined moderate and severe groups. In addition, the local public health policies and the lockdown laid down during the study sampling have limited access to patients who did not require hospitalization, thereby preventing the inclusion of participants with mild COVID-19 symptoms or even asymptomatic and uninfected or unvaccinated individuals. That restricted broader comparisons across the full spectrum of COVID-19 disease.

Despite these limitations, our findings provide important insights into the functional dysregulation of γδ T cells in COVID-19, although future investigations are necessary to prove the concepts observed and discussed here, and further understand the mechanisms underlying this selective functional impairment.

## Figures and Tables

**Table 1:** Sociodemographic and clinical characteristics by O^2^ support at hospitalization time.

	Overall (N = 44)	MWO_2_ (N = 15)	MO_2_ (N = 15)	SD (N = 14)	*p*-Value
Age; n (%)					0.405 ^(b)^
Median [Min, Max]	62.9 [26.7, 89.6]	52.9 [26.7, 84.7]	63.2 [31.4, 89.6]	65.9 [35.9, 82.0]	
Missing	1 (2.3%)	0 (0%)	0 (0%)	1 (7.1%)	
Gender at birth; n (%)					0.600 ^(a)^
Female	23 (52.3%)	7 (46.7%)	7 (46.7%)	9 (64.3%)	
Male	21 (47.7%)	8 (53.3%)	8 (53.3%)	5 (35.7%)	
Skin color; n (%)					0.053 ^(a)^
Black	5 (11.4%)	4 (26.7%)	1 (6.7%)	0 (0%)	
Brown	28 (63.6%)	8 (53.3%)	13 (86.7%)	7 (50.0%)	
White	9 (20.5%)	3 (20.0%)	1 (6.7%)	5 (35.7%)	
Missing	2 (4.5%)	0 (0%)	0 (0%)	2 (14.3%)	
Systemic hypertension; n (%)					1.000 ^(a)^
Yes	16 (36.4%)	5 (33.3%)	6 (40.0%)	5 (35.7%)	
Diabetes Mellitus; n (%)					0.129 ^(a)^
Yes	11 (25.0%)	5 (33.3%)	1 (6.7%)	5 (35.7%)	
Dyspnea; n (%)					0.082 ^(a)^
Yes	32 (72.7%)	9 (60.0%)	14 (93.3%)	9 (64.3%)	
Cough; n (%)					0.411^(a)^
Yes	26 (59.1%)	8 (53.3%)	11 (73.3%)	7 (50.0%)	
Oxygen saturation below 95%; n (%)					0.068 ^(a)^
Yes	19 (43.2%)	3 (20.0%)	9 (60.0%)	7 (50.0%)	
Fever ^(d)^; n (%)					0.229 ^(a)^
Yes	19 (43.2%)	6 (40.0%)	9 (60.0%)	4 (28.6%)	
Myalgia; n (%)					0.601 ^(a)^
Yes	13 (29.5%)	4 (26.7%)	6 (40.0%)	3 (21.4%)	
Anosmia; n (%)					0.594 ^(a)^
Yes	5 (11.4%)	3 (20.0%)	1 (6.7%)	1 (7.1%)	
Ageusia; n (%)					0.594 ^(a)^
Yes	5 (11.4%)	3 (20.0%)	1 (6.7%)	1 (7.1%)	
Coryza; n (%)					
Yes	5 (11.4%)	0 (0%)	3 (20.0%)	2 (14.3%)	
Diarrhea; n (%)					1.000 ^(a)^
Yes	1 (2.3%)	1 (6.7%)	0 (0%)	0 (0%)	
SOFA score					0.006 ^(c)^
Mean (Sd)	4.60 (3.66)	2.69 (2.84)	3.67 (2.26)	7.36 (4.07)	
Median [Min, Max]	4.00 [0, 13.0]	1.00 [0, 8.00]	3.00 [0, 7.00]	8.50 [0, 13.0]	
Missing	2 (4.5%)	2 (13.3%)	0 (0%)	0 (0%)	
Hospital outcome; n (%)					0.002 ^(a)^
Death	22 (50.0%)	3 (20.0%)	7 (46.7%)	12 (85.7%)	
Discharge	21 (47.7%)	12 (80.0%)	7 (46.7%)	2 (14.3%)	
Hospital transfer	1 (2.3%)	0 (0%)	1 (6.7%)	0 (0%)	

Data are expressed as absolute (relative) frequencies for nominal variables and as medians and interquartile ranges (IQRs) for continuous numerical variables. *p*-values were calculated using Fisher tests (a) for nominal variables, ANOVA (b) and Kruskal–Wallis (c) tests for continuous numerical variables, between MWO_2_, MO_2_ and SD groups; *p*-values < 0.05 were considered significant. (d) Fever was defined as an axillary temperature > 37.8 °C measured at hospital admission. Abbreviations: Min = minimum; Max = maximum; Sd = standard deviation; MWO_2_ = moderate cases without oxygen support; MO_2_ = moderate cases with non-invasive oxygen support; SD = severe disease; SOFA = sequential organ failure assessment.

## Data Availability

The datasets generated and/or analyzed during the current study are available from the corresponding author upon request. Public access to the data is restricted due to ethical and privacy considerations.

## Supplementary Materials

The following supporting information can be downloaded at: https://www.mdpi.com/article/10.3390/cells15111020/s1, Figure S1. Flow diagram of participant selection; Table S1. Flow cytometry panels used phenotypic and functional analysis of γδ T cells; Table S2. Laboratory findings at admission by ventilation assistance use at hospitalization time; Figure S2. Representative flow cytometry gating strategy to identify total γδ T cells and their subsets; Figure S3. Frequencies of γδ, Vδ1 and Vδ2 T cells analyzed among CD3^+^ cells, considering sex and age, in moderate and severe COVID-19 in-patients; Figure S4. In vitro degranulation and cytokine production by total γδ T cells; Figure S5. γδ^+^Vδ1^+^, γδ^+^Vδ2^+^, and γδ^+^Vδ1^−^Vδ2^−^ frequencies in CD3^+^ PBMCs among MWO_2_ (N = 14), MO_2_ (N = 14) and SD (N = 12) groups; Figure S6. Flow cytometric distribution of TNF-α^+^, CD107a^+^ and IFN-γ^+^ expression frequencies, and CD107a^+^ and IFN-γ^+^ MFI according to clinical presentation after in vitro stimulation; Figure S7. Flow cytometric distribution of IL-1βR^+^ and IL-23R^+^ expression and CD161^+^, IL-1βR^+^ and IL-23R^+^ MFI and according to clinical presentation after in vitro stimulation; Figure S8. t-SNE analysis of functional marker expression in γδ^+^Vδ2^+^ cells; Figure S9. γδ^+^Vδ1^+^, γδ^+^Vδ2^+^, and γδ^+^Vδ1^−^Vδ2^−^ frequencies ex vivo (top) and after in vitro stimulation (bottom) in γδ T cell subsets according to clinical outcomes.
