# Supplementary material for: Functional Profile of γδ T Cells in Severe and Moderate COVID-19: A Brazilian Cross-Sectional Study
Source: Cells. 2026 Jun 1;15(11):1020. doi: 10.3390/cells15111020 (PMC13256624; doi:10.3390/cells15111020)
Supplement: Supplementary file 1 [file cells-15-01020-s001.zip › cells-4204472-supplementary.pdf]

cells

MDPI

Article

Functional Profile of γδ T Cells in Severe and Moderate COVID-19: A Brazilian Cross-Sectional Study

Andressa da Silva Cazote [1], Glenda Domingos Mascarenhas [1], Hugo Perazzo [2], Kim Mattos Geraldo [2], Maria Pia Diniz Ribeiro [2], Juliana Arruda de Matos [2], Pedro Emmanuel Alvarenga Americano do Brasil [2], Sandra Wagner Cardoso [2], Beatriz Grinsztejn [2], Valdiléa Gonçalves Veloso [2], Cynthia Machado Cascabulho [3], José Henrique Pilotto [1], Diogo Gama Caetano [1], Milena Neira Guimarães Goulart [1], Nathalia Beatriz Ramos de Sá [1], Dalziza Victalina de Almeida [1], Fernanda Heloise Côrtes [1], Mariza Gonçalves Morgado [1] and Carmem Beatriz Wagner Giacoia-Gripp [1,*]

[1] Laboratório de AIDS & Imunologia Molecular, Instituto Oswaldo Cruz (IOC), FIOCRUZ, Rio de Janeiro 21040-360, Brazil; andressacazote@gmail.com (A.d.S.C.); gdomingos@biof.ufrj.br (G.D.M.); pilotto@ioc.fiocruz.br (J.H.P.); diogo.caetano91@gmail.com (D.G.C.); milenanggoulart@gmail.com (M.N.G.G.); nathalia.ramos@ioc.fiocruz.br (N.B.R.d.S.); dalziza@ioc.fiocruz.br (D.V.d.A.); fheloise@ioc.fiocruz.br (F.H.C.); mmorgado@ioc.fiocruz.br (M.G.M.)

[2] Instituto Nacional de Infectologia Evandro Chagas (INI), FIOCRUZ, Rio de Janeiro 21040-360, Brazil; perazzohugo@gmail.com (H.P.); kim.geraldo@ini.fiocruz.br (K.M.G.); mariapia.diniz@ini.fiocruz.br (M.P.D.R.); juliana.matos@ini.fiocruz.br (J.A.d.M.); pedro.brasil@fiocruz.br (P.E.A.A.d.B.); sandra.wagner@ini.fiocruz.br (S.W.C.); beatriz.grinsztejn@gmail.com (B.G.); valdilea.veloso@gmail.com (V.G.V.)

[3] Flow Cytometry Platform—Multiparametric Analysis Unit, Oswaldo Cruz Institute, Rio de Janeiro 21040-360, Brazil; cynthiac@ioc.fiocruz.br

* Correspondence: cbwggripp@gmail.com

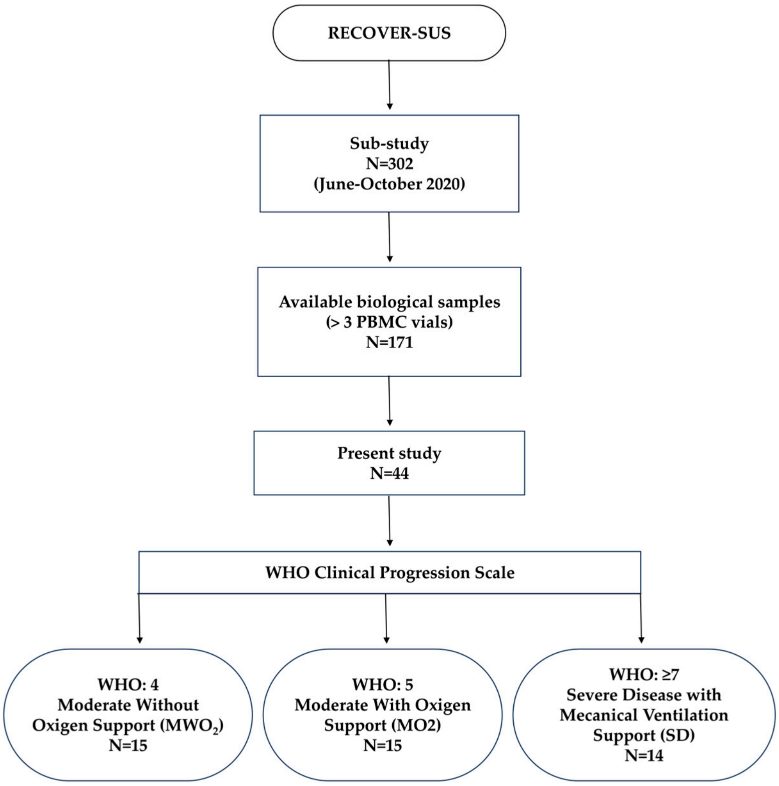

Figure S1. Flow diagram of participant selection. A total of 302 unvaccinated in-patients with confirmed SARS-CoV-2 infection, admitted between June and October 2020 to the RECOVER-SUS cohort at INI/FIOCRUZ, were initially enrolled in an immunological, virological, and genetic substudy upon written informed consent. From 171 patients with enough biological material and available (≥3 PBMC vials), 44 individuals were selected for the present cross-sectional study. Patients

were stratified according to the WHO Clinical Progression Scale into three groups: moderate disease without oxygen support (MWO_2, N = 15), moderate disease with oxygen support (MO_2, N = 15), and severe disease requiring mechanical ventilation (SD, N = 14).

Table S1. Flow cytometry panels used phenotypic and functional analysis of γδ T cells.

Panels	Antibody	Clone	Conjugated- fluorophore	Enterprise
1,2	anti-TCRγδ	B1	PE-CF594	BD Biosciences
	anti-Vδ1	R9.12	PC7	Beckman Coulter
	anti-Vδ2	B6	APC-Fire750	BioLegend
	anti-CD27	M-T271	BV605	BD Biosciences
	anti-CD161	HP-3G10	PerCP-Cy-5.5	BioLegend
1	anti-TRAIL	RIK2	BV421	BD Biosciences
	anti-IFN-γ	4S.B3	PE	BD Biosciences
	anti-TNF-α	MAb11	APC	BD Biosciences
2	anti-IL-1R		PE	R&D Systems
	anti-IL-23R	218213	APC	R&D Systems
	anti-IL-17	N49-653	BV421	BD Biosciences

PE: Phycoerythrin; CF: Cyanine-based Fluorescent; PC: Phycoerythrin-Cyanine; APC: Allophycocyanin; BV: Brilliant Violet; PerCP: Peridinin-Chlorophyll-Protein; Cy: Cyanine.

Table S2. Laboratory findings at admission by ventilation assistance use at hospitalization time.

	Overall (N=44)	MWO_2 (N=15)	MO_2 (N=15)	SD (N=14)	*p*-Value
**HIV positive; n (%)**					1.000[(a)]
Positive	7 (15.9%)	3 (20.0%)	2 (13.3%)	2 (14.3%)	
**Chronic obstructive pulmonary disease; n (%)**					0.202[(a)]
Yes	4 (9.1%)	0 (0%)	3 (20.0%)	1 (7.1%)	
**Previous stroke; n (%)**					0.302[(a)]
Yes	3 (6.8%)	0 (0%)	1 (6.7%)	2 (14.3%)	
**Heart failure; n (%)**					0.318[(a)]
Yes	1 (2.3%)	0 (0%)	0 (0%)	1 (7.1%)	
**Coronary artery disease; n (%)**					1.000[(a)]
Yes	1 (2.3%)	0 (0%)	1 (6.7%)	0 (0%)	
**Chronic kidney disease; n (%)**					0.443[(a)]
Yes	4 (9.1%)	0 (0%)	2 (13.3%)	2 (14.3%)	
**Rheumatic disease; n (%)**					0.108[(a)]
Yes	4 (9.1%)	1 (6.7%)	0 (0%)	3 (21.4%)	
**Previous bariatric surgery; n (%)**					0.334[(a)]
Yes	9 (20.5%)	2 (13.3%)	2 (13.3%)	5 (35.7%)	

**Current smoking; n (%)**					0.302[(a)]
Yes	4 (9.1%)	1 (6.7%)	3 (20.0%)	0 (0%)	
**Previous smoking; n (%)**					0.524[(a)]
Yes	3 (6.8%)	0 (0%)	2 (13.3%)	1 (7.1%)	
**Active tuberculosis; n (%)**					1.000[(a)]
Yes	4 (9.1%)	2 (13.3%)	1 (6.7%)	1 (7.1%)	
**Treated tuberculosis; n (%)**					1.000[(a)]
Yes	1 (2.3%)	0 (0%)	1 (6.7%)	0 (0%)	
**Length of hospital stay; n (%)**					0.588[(a)]
[0,7]	16 (36.4%)	5 (33.3%)	4 (26.7%)	7 (50.0%)	
[7,14]	16 (36.4%)	5 (33.3%)	8 (53.3%)	3 (21.4%)	
[14,21]	3 (6.8%)	2 (13.3%)	1 (6.7%)	0 (0%)	
[21,28]	2 (4.5%)	1 (6.7%)	0 (0%)	1 (7.1%)	
[28,35]	2 (4.5%)	0 (0%)	1 (6.7%)	1 (7.1%)	
[35,42]	2 (4.5%)	1 (6.7%)	1 (6.7%)	0 (0%)	
[42,49]	1 (2.3%)	1 (6.7%)	0 (0%)	0 (0%)	
[49,56]	1 (2.3%)	0 (0%)	0 (0%)	1 (7.1%)	
[56,63]	0 (0%)	0 (0%)	0 (0%)	0 (0%)	
[63,70]	0 (0%)	0 (0%)	0 (0%)	0 (0%)	
[70,77]	1 (2.3%)	0 (0%)	0 (0%)	1 (7.1%)	
**Leukocytes; n (%)**					0.691[(b)]
Mean (Sd)	14000 (6340)	13000 (7980)	14000 (5150)	15100 (5710)	
Median [Min, Max]	14300 [3670, 30200]	11600 [3780, 30200]	15000 [3670, 23300]	16800 [4600, 23000]	
Missing	1 (2.3%)	0 (0%)	1 (6.7%)	0 (0%)	
**Lymphocytes; n (%)**					0.584[(c)]
Mean (Sd)	1580 (979)	1530 (824)	1410 (939)	1800 (1180)	
Median [Min, Max]	1240 [11.4, 3920]	1240 [11.4, 3540]	1130 [147, 3160]	1330 [691, 3920]	
Missing	1 (2.3%)	0 (0%)	1 (6.7%)	0 (0%)	
**Platelets; n (%)**					0.713[(c)]
Mean (Sd)	207000 (199000)	233000 (245000)	216000 (161000)	170000 (185000)	
Median [Min, Max]	198000 [167, 662000]	198000 [167, 609000]	237000 [227, 454000]	171000 [185, 662000]	
Missing	1 (2.3%)	0 (0%)	1 (6.7%)	0 (0%)	
**Creatinine; n (%)**					0.023[(c)]
Mean (Sd)	1.59 (1.34)	1.54 (1.50)	1.02 (0.369)	2.25 (1.59)	
Median [Min, Max]	1.09 [0.460, 6.66]	1.05 [0.710, 6.42]	0.980 [0.460, 1.88]	1.53 [0.710, 6.66]	
Missing	2 (4.5%)	0 (0%)	1 (6.7%)	1 (7.1%)	
**Amylase; n (%)**					0.207[(c)]
Mean (Sd)	84.9 (73.4)	85.8 (56.6)	55.7 (30.9)	115 (105)	
Median [Min, Max]	64.0 [14.0, 371]	72.0 [14.0, 212]	55.5 [14.0, 117]	75.0 [18.0, 371]	
Missing	5 (11.4%)	3 (20.0%)	1 (6.7%)	1 (7.1%)	
**Lipase; n (%)**					0.223[(c)]

Mean (Sd)	58.4 (64.2)	57.2 (53.4)	44.6 (62.1)	74.5 (76.0)	
Median [Min, Max]	36.0 [9.00, 266]	36.0 [20.0, 181]	31.0 [9.00, 256]	48.0 [12.0, 266]	
Missing	5 (11.4%)	3 (20.0%)	1 (6.7%)	1 (7.1%)	
**Alanine aminotransferase; n (%)**					0.181[(c)]
Mean (Sd)	49.1 (55.3)	32.5 (30.6)	43.9 (23.0)	71.8 (87.9)	
Median [Min, Max]	31.0 [8.00, 318]	18.0 [10.0, 116]	46.0 [8.00, 81.0]	31.0 [8.00, 318]	
Missing	3 (6.8%)	2 (13.3%)	0 (0%)	1 (7.1%)	
**Aspartate aminotransferase; n (%)**					0.512[(c)]
Mean (Sd)	46.8 (39.8)	43.7 (42.9)	41.7 (22.0)	55.8 (52.4)	
Median [Min, Max]	30.0 [10.0, 204]	28.0 [10.0, 153]	30.0 [15.0, 80.0]	37.0 [10.0, 204]	
Missing	3 (6.8%)	2 (13.3%)	0 (0%)	1 (7.1%)	
**Bilirubin; n (%)**					0.302[(c)]
Mean (Sd)	0.560 (0.347)	0.498 (0.198)	0.458 (0.190)	0.742 (0.518)	
Median [Min, Max]	0.470 [0.140, 1.87]	0.470 [0.270, 0.840]	0.380 [0.240, 0.820]	0.560 [0.140, 1.87]	
Missing	3 (6.8%)	2 (13.3%)	0 (0%)	1 (7.1%)	
**D-Dimer (Finecare); n (%)**					0.389[(c)]
Mean (Sd)	5.84 (4.71)	5.21 (3.89)	4.36 (4.29)	7.76 (5.45)	
Median [Min, Max]	5.60 [0.300, 20.0]	5.65 [0.400, 10.0]	1.95 [0.300, 10.6]	8.86 [0.300, 20.0]	
Missing	13 (29.5%)	5 (33.3%)	5 (33.3%)	3 (21.4%)	
**C-reactive protein; n (%)**					0.645[(c)]
Mean (Sd)	12.8 (9.72)	12.7 (9.60)	10.9 (8.77)	15.1 (11.0)	
Median [Min, Max]	11.8 [1.10, 39.9]	12.3 [1.22, 25.4]	9.68 [1.39, 33.1]	11.8 [1.10, 39.9]	
Missing	3 (6.8%)	1 (6.7%)	1 (6.7%)	1 (7.1%)	
**Erythrocyte sedimentation rate; n (%)**					0.496[(b)]
Mean (Sd)	84.9 (38.6)	89.9 (39.8)	73.3 (30.9)	90.5 (43.8)	
Median [Min, Max]	92.0 [5.00, 150]	100 [9.00, 150]	80.0 [35.0, 120]	110 [5.00, 150]	
Missing	9 (20.5%)	4 (26.7%)	4 (26.7%)	1 (7.1%)	
**Procalcitonin; n (%)**					0.242[(c)]
Mean (Sd)	6.55 (19.4)	6.57 (20.3)	1.77 (4.66)	12.1 (27.7)	
Median [Min, Max]	0.470 [0.100, 94.6]	0.545 [0.140, 70.9]	0.225 [0.100, 17.8]	0.685 [0.100, 94.6]	
Missing	6 (13.6%)	3 (20.0%)	1 (6.7%)	2 (14.3%)	
**Ferritin; n (%)**					0.199[(c)]
Mean (Sd)	559 (455)	385 (381)	797 (581)	472 (325)	
Median [Min, Max]	652 [1.49, 1500]	372 [1.49, 850]	912 [1.50, 1500]	622 [1.50, 906]	
Missing	20 (45.5%)	9 (60.0%)	7 (46.7%)	4 (28.6%)	
**Troponin; n (%)**					0.092[(c)]

Mean (Sd)	0.495 (2.41)	0.0215 (0.0138)	0.0784 (0.187)	1.28 (3.99)	
Median [Min, Max]	0.0300 [0, 15.0]	0.0290 [0, 0.0300]	0.0295 [0, 0.720]	0.0300 [0, 15.0]	
Missing	5 (11.4%)	4 (26.7%)	1 (6.7%)	0 (0%)	
**Glycated hemoglobin (HbA1c); n (%)**					0.725[(c)]
Mean (Sd)	8.82 (10.6)	13.8 (20.4)	6.52 (0.928)	7.21 (3.02)	
Median [Min, Max]	6.05 [2.60, 55.0]	5.40 [2.60, 55.0]	6.05 [5.80, 8.10]	6.40 [3.90, 12.7]	
Missing	22 (50.0%)	9 (60.0%)	9 (60.0%)	4 (28.6%)	
**Prothrombin Time; n (%)**					0.058[(c)]
Mean (Sd)	1.15 (0.189)	1.07 (0.0824)	1.11 (0.157)	1.26 (0.238)	
Median [Min, Max]	1.07 [0.950, 1.78]	1.06 [0.970, 1.19]	1.06 [0.950, 1.50]	1.21 [0.960, 1.78]	
Missing	5 (11.4%)	3 (20.0%)	1 (6.7%)	1 (7.1%)	
**Partial Thromboplastin Time; n (%)**					0.827[(c)]
Mean (Sd)	32.3 (18.0)	29.3 (8.11)	35.0 (28.1)	32.3 (11.7)	
Median [Min, Max]	27.5 [18.0, 127]	26.0 [22.0, 50.9]	27.0 [21.4, 127]	30.3 [18.0, 55.7]	
Missing	6 (13.6%)	3 (20.0%)	2 (13.3%)	1 (7.1%)	
**Urea; n (%)**					0.060[(c)]
Mean (Sd)	76.8 (58.3)	69.3 (62.1)	58.2 (48.8)	107 (56.0)	
Median [Min, Max]	62.0 [16.0, 221]	40.0 [18.3, 221]	37.7 [16.0, 213]	107 [18.3, 203]	
Missing	1 (2.3%)	0 (0%)	0 (0%)	1 (7.1%)	

Data are expressed as absolute (relative) frequencies for nominal variables and as medians and interquartile ranges (IQRs) for continuous numerical variables. *p*-values were calculated using Fisher tests[(a)] for nominal variables, ANOVA[(b)] and Kruskal–Wallis[(c)] tests for continuous–numerical variables, between MWO_2, MO_2 and SD groups; *p*-values<0.05 were considered significant.
Abbreviations: HIV = Human Immunodeficiency Virus; Min = minimum; Max = maximum; Sd = Standard deviation; MWO_2 = moderate cases without oxygen support; MO_2 = moderate with oxygen support; SD = severe disease.

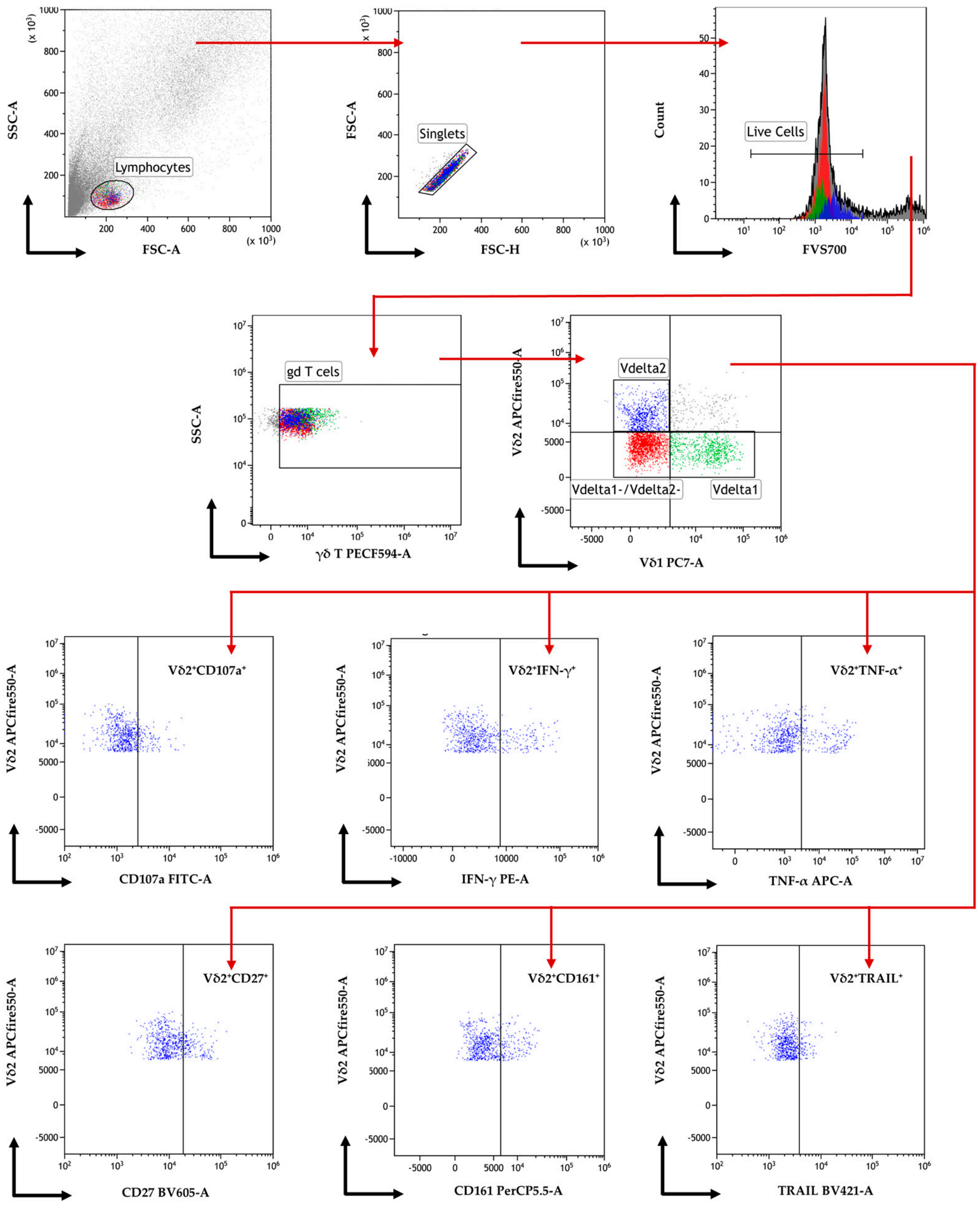

Figure S2. Representative flow cytometry gating strategy to identify total γδ T cells and their subsets. After initial lymphocyte and singlet selection, γδ T cells were defined based on TCRγδ expression and further the subsets were defined based on Vδ1 and Vδ2 expression into Vδ1$^+$, Vδ2$^+$, and Vδ1$^-$Vδ2$^-$ subpopulations. Within each γδ T-cell subset, each molecule was subsequently assessed. Gate positions were defined based on unstained and FMO controls.

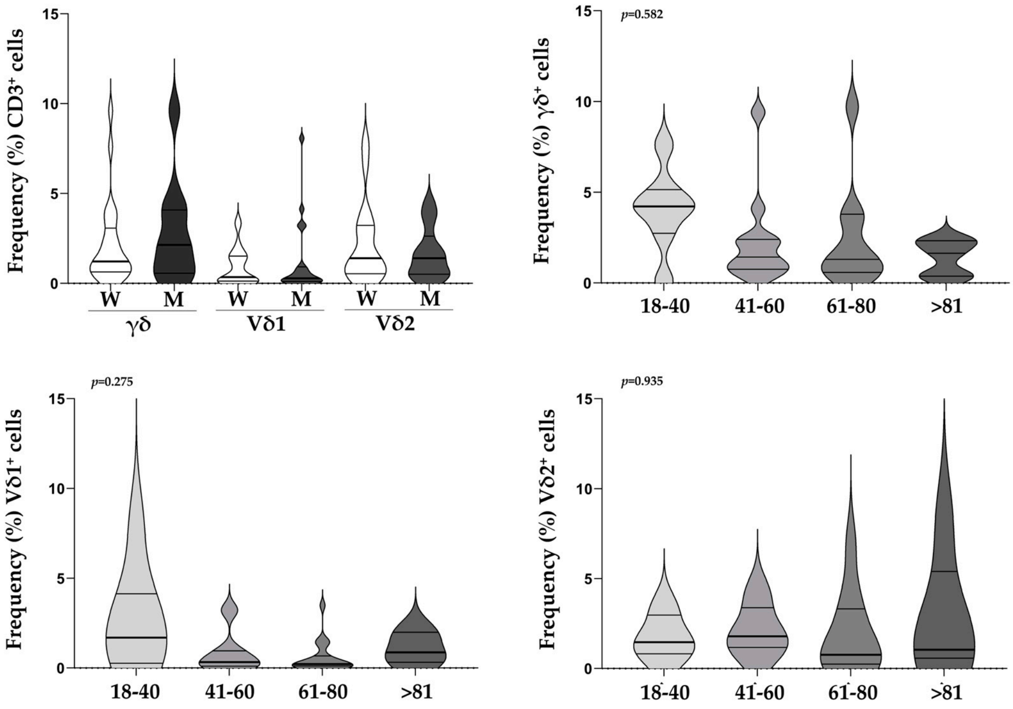

Figure S3. Frequencies of γδ, Vδ1 and Vδ2 T cells analyzed among CD3+ cells, considering sex and age, in moderate and severe COVID-19 in-patients. Violine plots shows γδ, Vδ1 and Vδ2 T cells frequencies compared between women (W, represented in white, N=24) and men (M, represented in graphite, N=20). Also, violine plots shows the frequencies of γδ, Vδ1 and Vδ2 T cells compared across age categories (18-40, 41-60, 61-80, >81 years, represented in gray scale from lighter to darker, respectively). Medians and interquartile ranges are represented by horizontal bars in graphs. Clinical study groups were analyzed by Kruskal-Wallis test and *p*-values are indicated in the superior left corner at each graph. Wilcoxon matched-pairs signed-rank test or Dunn's test *p*-values <0.05 were considered significant.

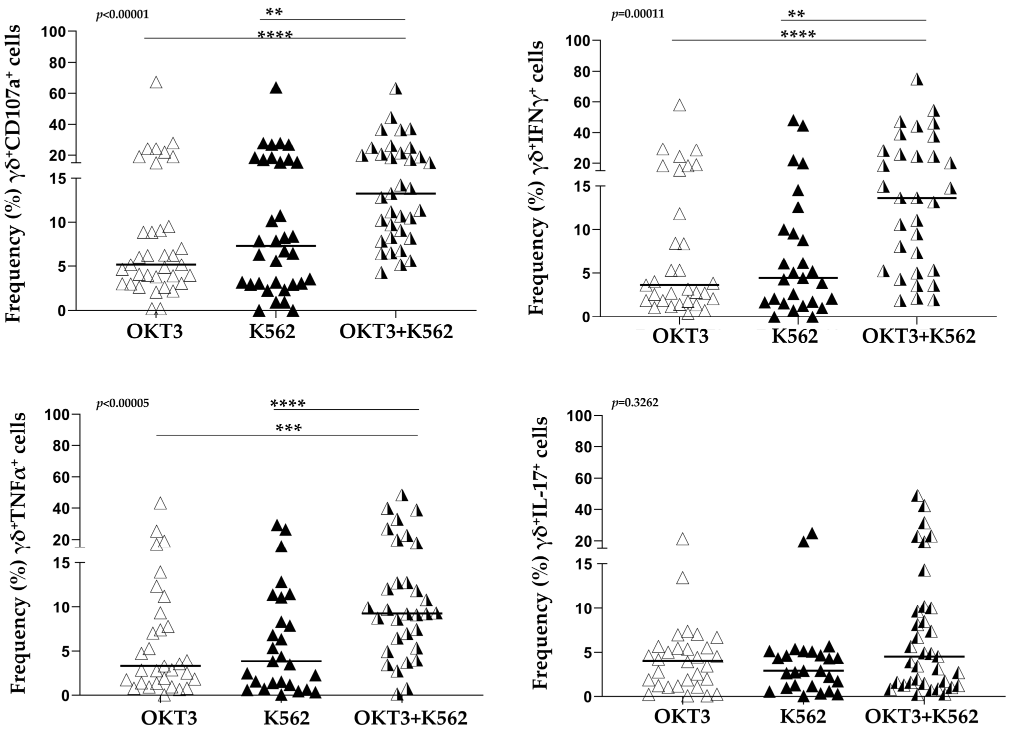

Figure S4. *In vitro* degranulation and cytokine production by total γδ T cells. *In vitro* expression of degranulation marker CD107a and intracellular production of IFNγ, TNFα, and IL-17 by total γδ T cells co-cultured with K562 cell line in the presence of OKT3 (half-filled triangles), compared with stimulation with OKT3 alone (open triangles) and K562 alone (filled triangles). Medians are represented by horizontal bars. Stimuli were compared using Friedman test, followed by post-hoc comparison test with Wilcoxon matched-pairs signed-rank test. *p*-values <0.05 were considered significant and are represented as: ** $p < 0.01$; *** $p < 0.005$; and **** $p < 0.0005$.

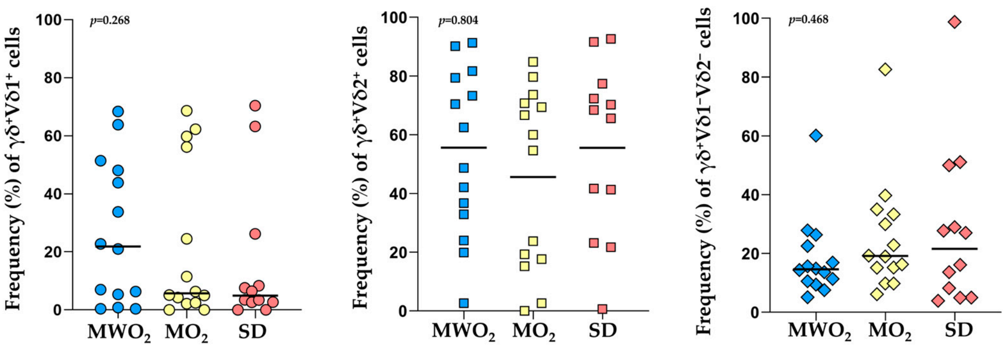

Figure S5. γδ+Vδ1+, γδ+Vδ2+, and γδ+Vδ1-Vδ2- frequencies in CD3+ PBMCs among MWO_2 (N=14), MO_2 (N=14) and SD (N=12) groups. Medians are represented by horizontal bars. MWO_2 group is represented in blue. MO_2 group is presented in yellow. SD group is represented in red. Vδ1+ cells are represented in circles. Vδ2+ are represented in squares. Vδ1-/Vδ2- cells are represented in diamond shapes. Clinical study groups were analyzed by Kruskal-Wallis test and *p*-values are indicated in the superior left corner at each graph. Dunn's test *p*-values <0.05 were considered

significant.. MWO2 patients are represented in blue; MO2 patients are represented in yellow; SD patients are represented in red.

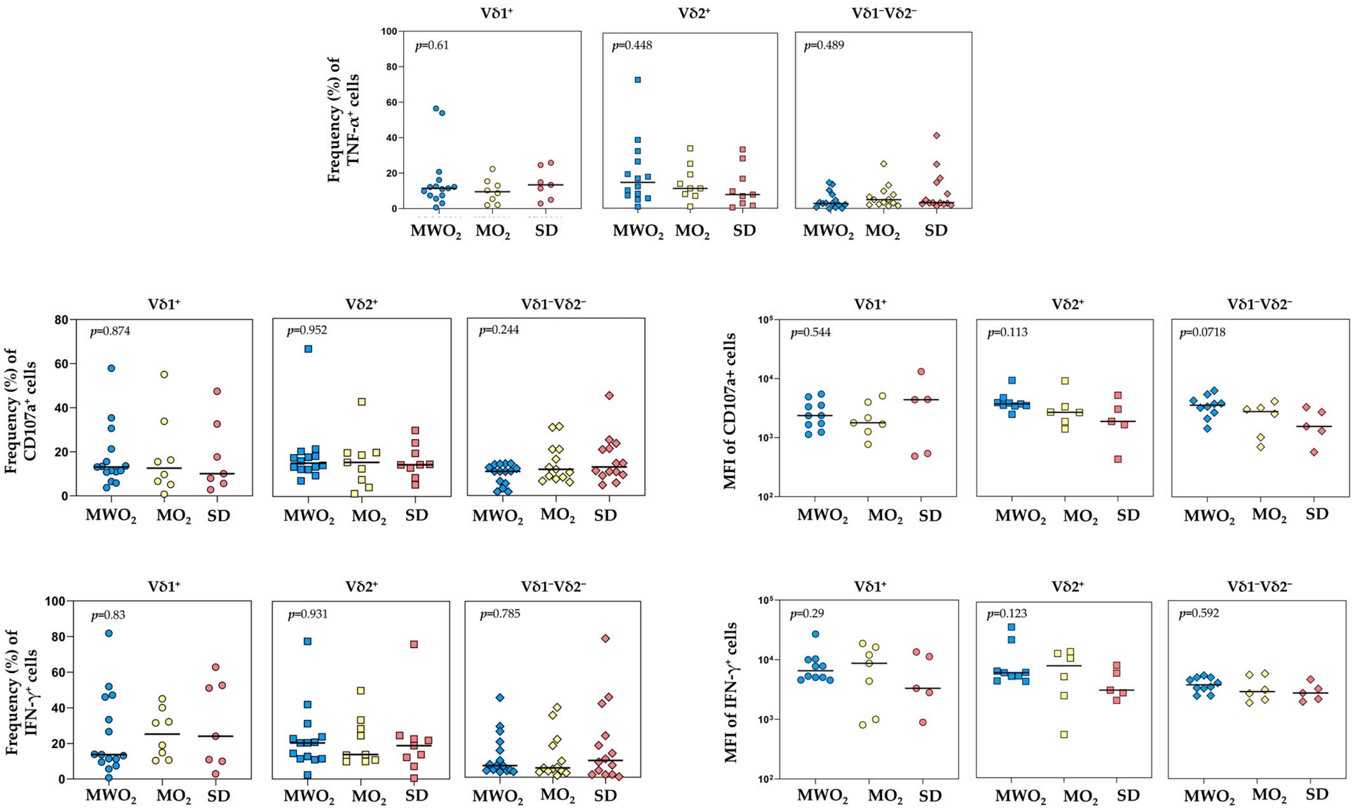

Figure S6. Flow cytometric distribution of TNF-α^+, CD107a$^+$ and IFN-γ^+ expression frequencies, and CD107a$^+$ and IFN-γ^+ MFI according to clinical presentation after *in vitro* stimulation. Medians are represented by horizontal bars. MWO2 group is represented in blue. MO2 group is presented in yellow. SD group is represented in red. Vδ1$^+$ cells are represented in circles. Vδ2$^+$ are represented in squares. Vδ1$^-$Vδ2$^-$ cells are represented in diamond shapes. Clinical study groups were analyzed by Kruskal-Wallis test and *p*-values are indicated in the superior left corner at each graph. Dunn's test *p*-values <0.05 were considered significant. MWO2 patients are represented in blue; MO2 patients are represented in yellow; SD patients are represented in red.

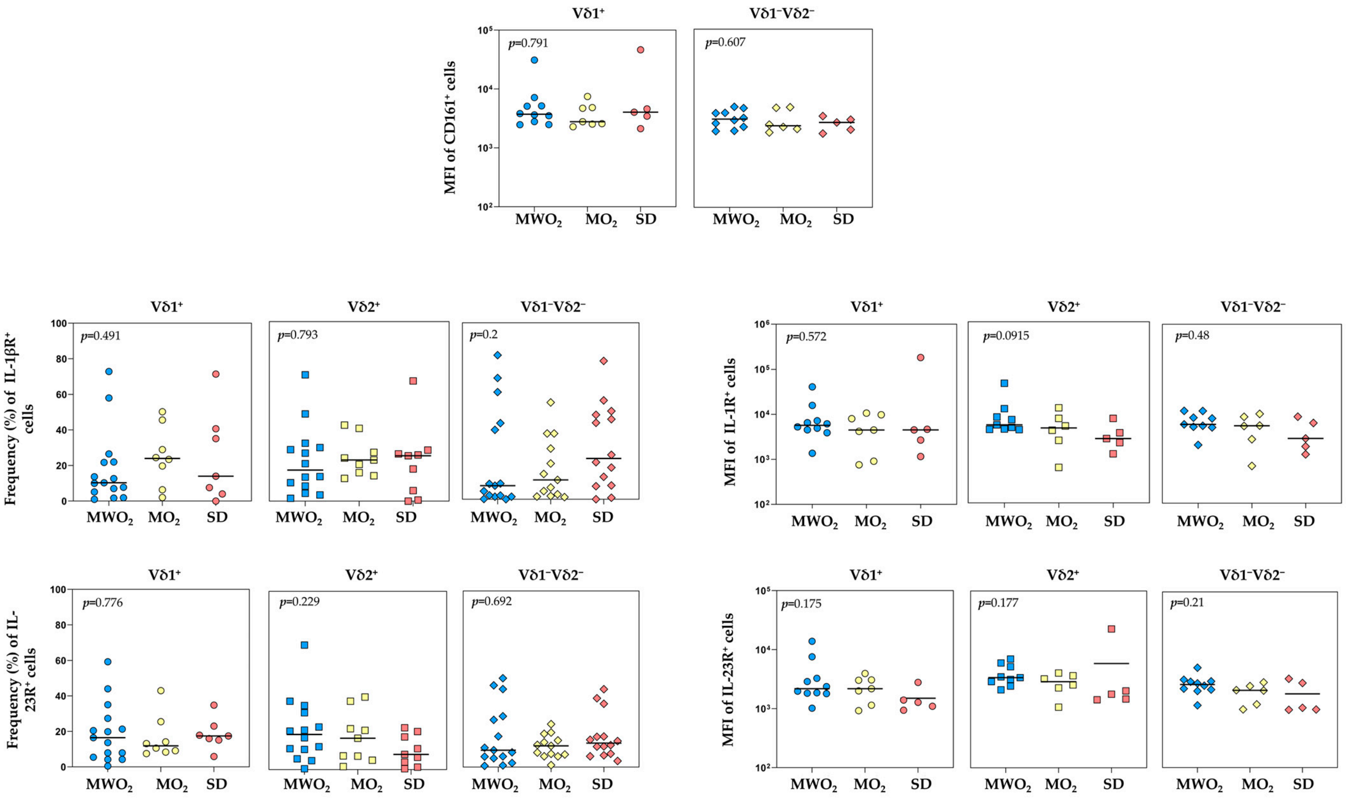

Figure S7. Flow cytometric distribution of IL-1βR$^+$ and IL-23R$^+$ expression and CD161$^+$, IL-1βR$^+$ and IL-23R$^+$ MFI and according to clinical presentation after *in vitro* stimulation. Medians are represented by horizontal bars. MWO_2 group is represented in blue. MO_2 group is presented in yellow. SD group is represented in red. Vδ1$^+$ cells are represented in circles. Vδ2$^+$ are represented in squares. Vδ1$^-$Vδ2$^-$ cells are represented in diamond shapes. Clinical study groups were analyzed by Kruskal-Wallis test and *p*-values are indicated in the superior left corner at each graph. Dunn's test *p*-values <0.05 were considered significant.

MWO_2 patients are represented in blue; MO_2 patients are represented in yellow; SD patients are represented in red.

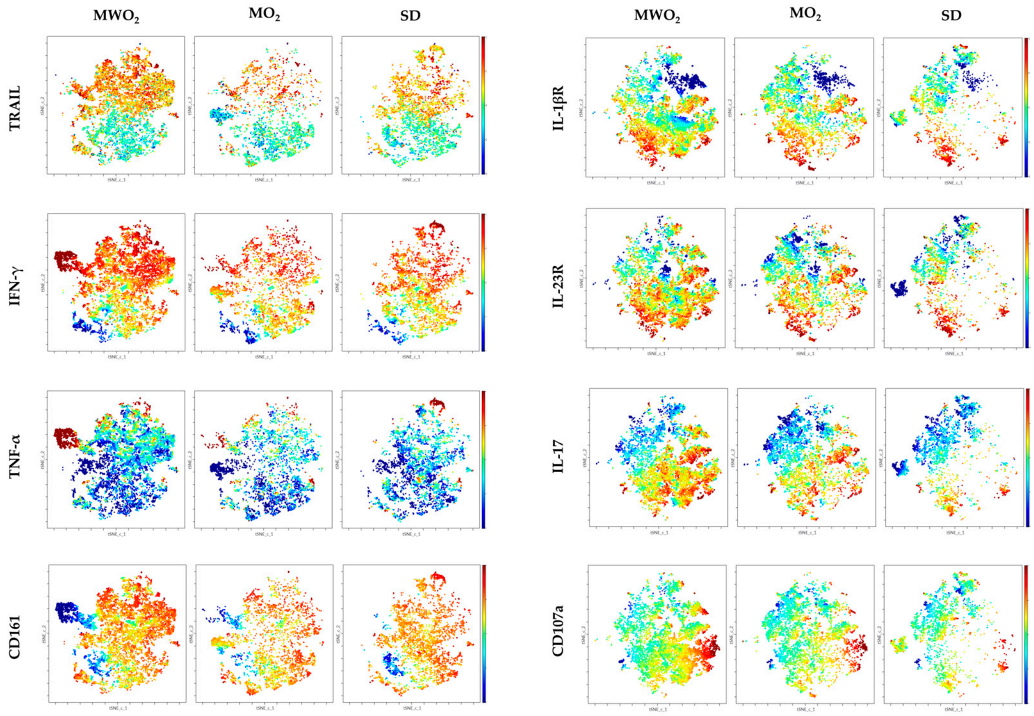

Figure S8. t-SNE analysis of functional marker expression in $\gamma\delta^{+}V\delta2^{+}$ cells. t-SNE plots of concatenated $\gamma\delta^{+}V\delta2^{+}$ cells samples including all groups. Cells are displayed according to severity groups: MWO_2, MO_2, and SD (columns). Each dot represents a single cell, and colors indicate normalized expression levels (blue = low, red = high). Rows show the expression of functional and phenotypic markers in the following order: TRAIL, IFN-γ, TNF-α, CD161, IL-1R, IL-23R, IL-17, and CD107a. The same t-SNE coordinates were applied across all panels to enable direct comparison of marker expression patterns among $\gamma\delta$ T cell subsets.

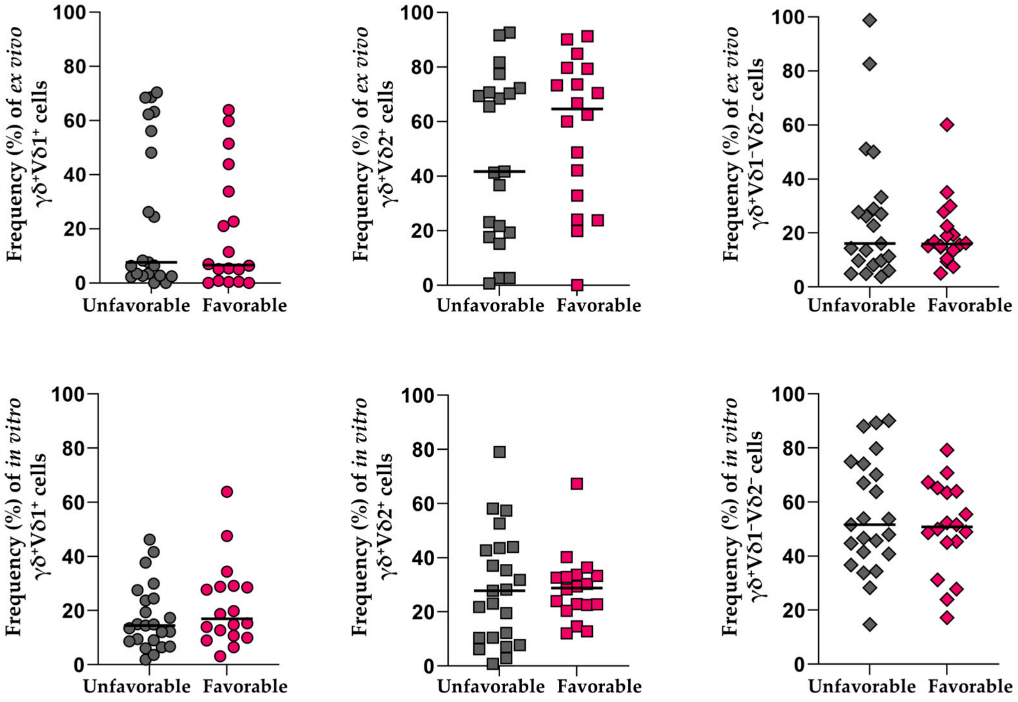

Figure S9. $\gamma\delta^+V\delta1^+$, $\gamma\delta^+V\delta2^+$, and $\gamma\delta^+V\delta1^-V\delta2^-$ frequencies *ex vivo* (top) and after *in vitro* stimulation (bottom) in γδ T-cell subsets according to clinical outcomes. Medians are represented by horizontal bars. Unfavorable outcome is represented in gray. Favorable outcome is represented in pink. $V\delta1^+$ cells are represented in circles. $V\delta2^+$ are represented in squares. $V\delta1^-V\delta2^-$ cells are represented in diamond shapes. Groups were compared using Friedman test, followed by post-hoc comparison tests with Wilcoxon matched-pairs signed-rank Test. *P*-values < 0.05 were considered significant.
