# Supplementary figures and images for: Functional Profile of γδ T Cells in Severe and Moderate COVID-19: A Brazilian Cross-Sectional Study
Source: Cells. 2026 Jun 1;15(11):1020. doi: 10.3390/cells15111020 (PMC13256624; doi:10.3390/cells15111020)

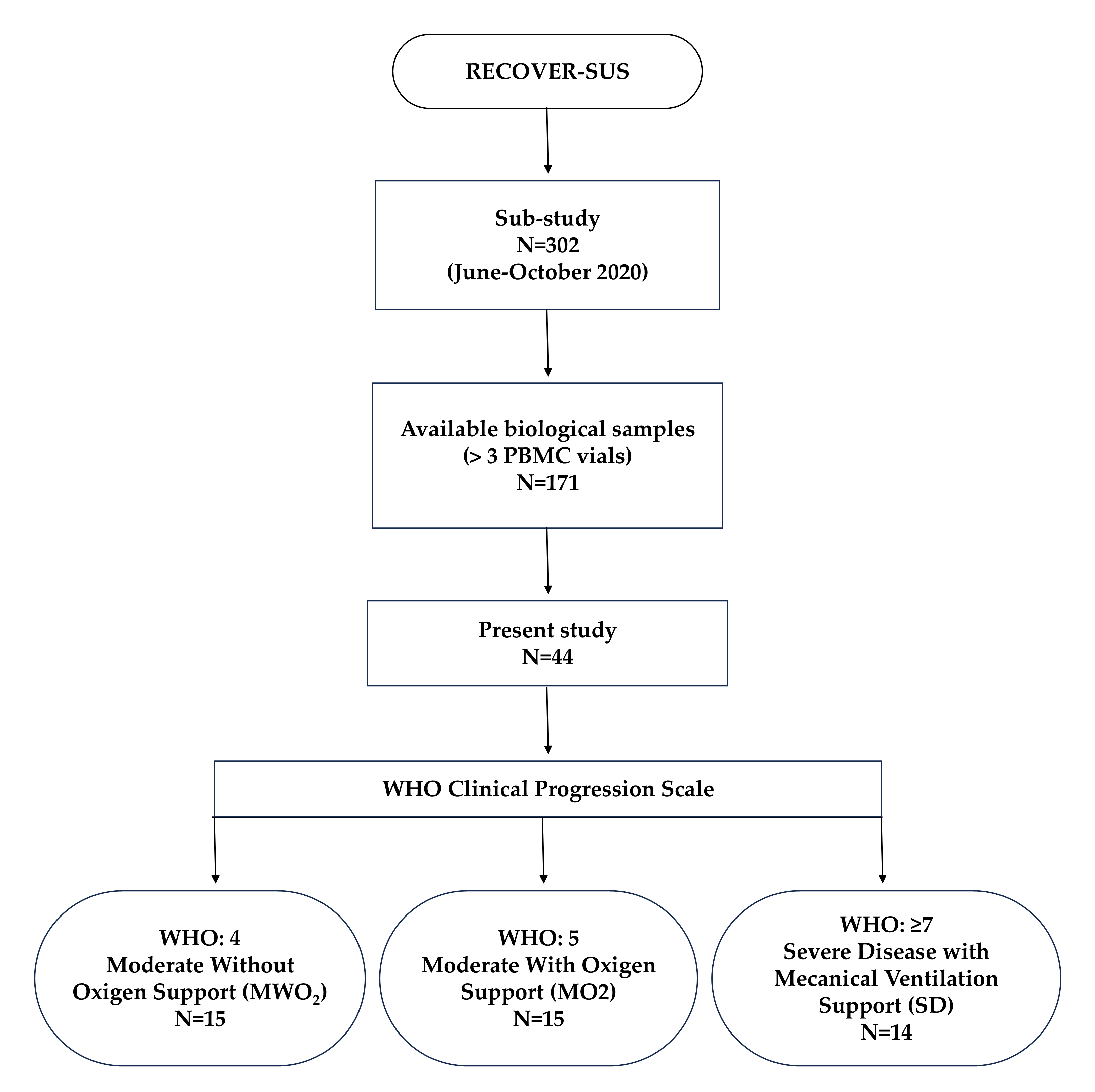

Supplement: Supplementary file 1 [file cells-15-01020-s001.zip › Figure S1.TIF]

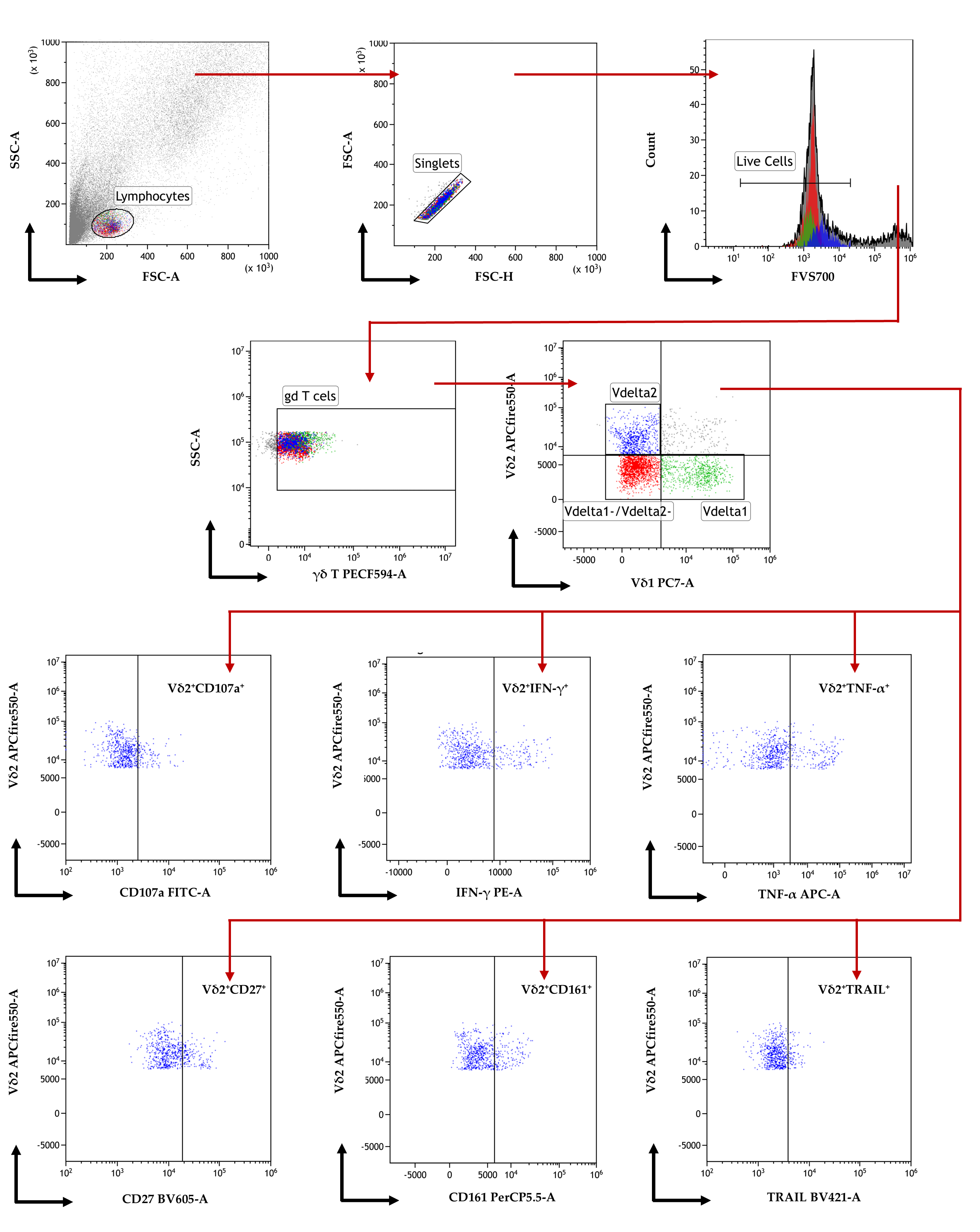

Supplement: Supplementary file 1 [file cells-15-01020-s001.zip › Figure S2.TIF]

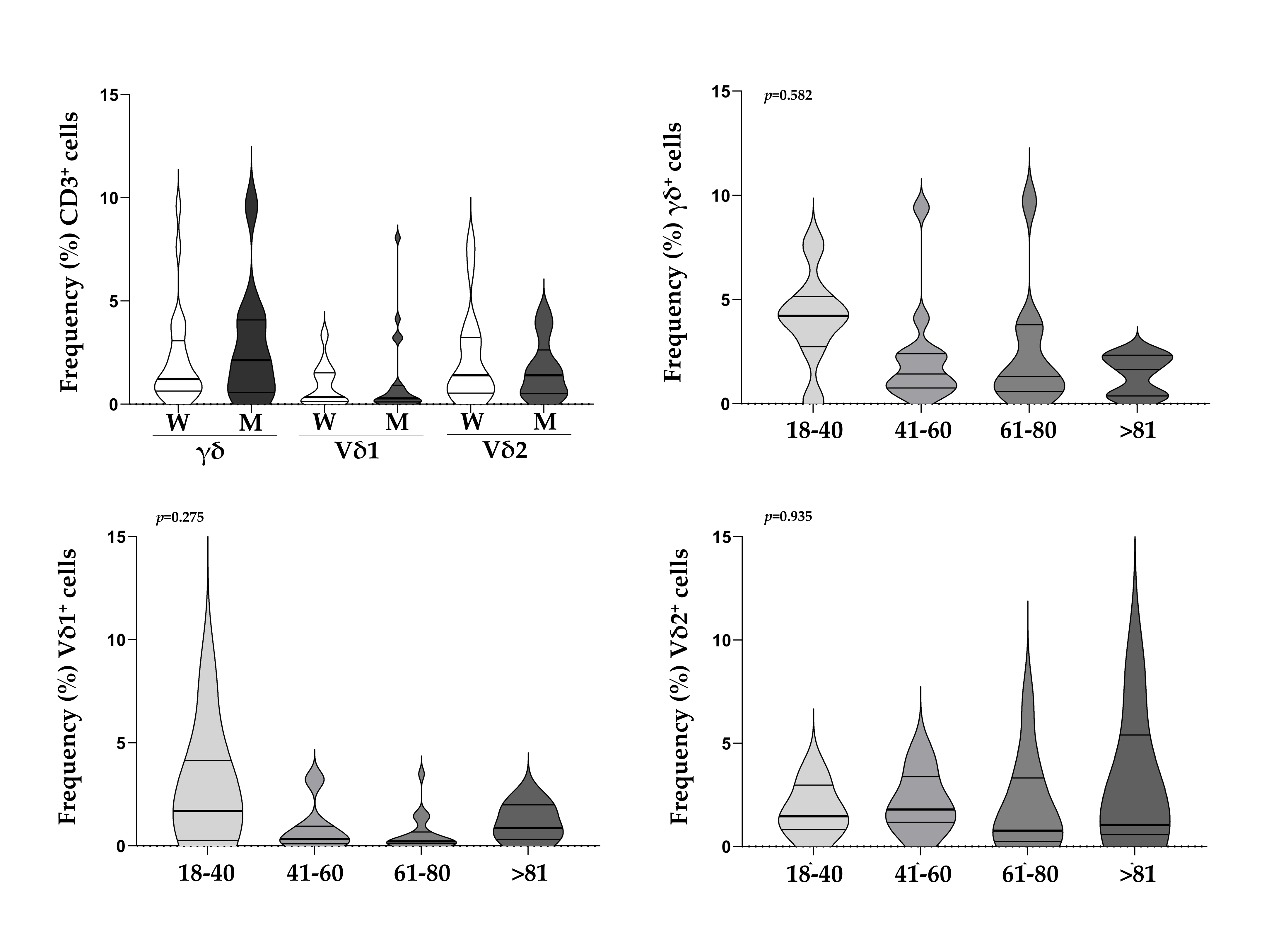

Supplement: Supplementary file 1 [file cells-15-01020-s001.zip › Figure S3.TIF]

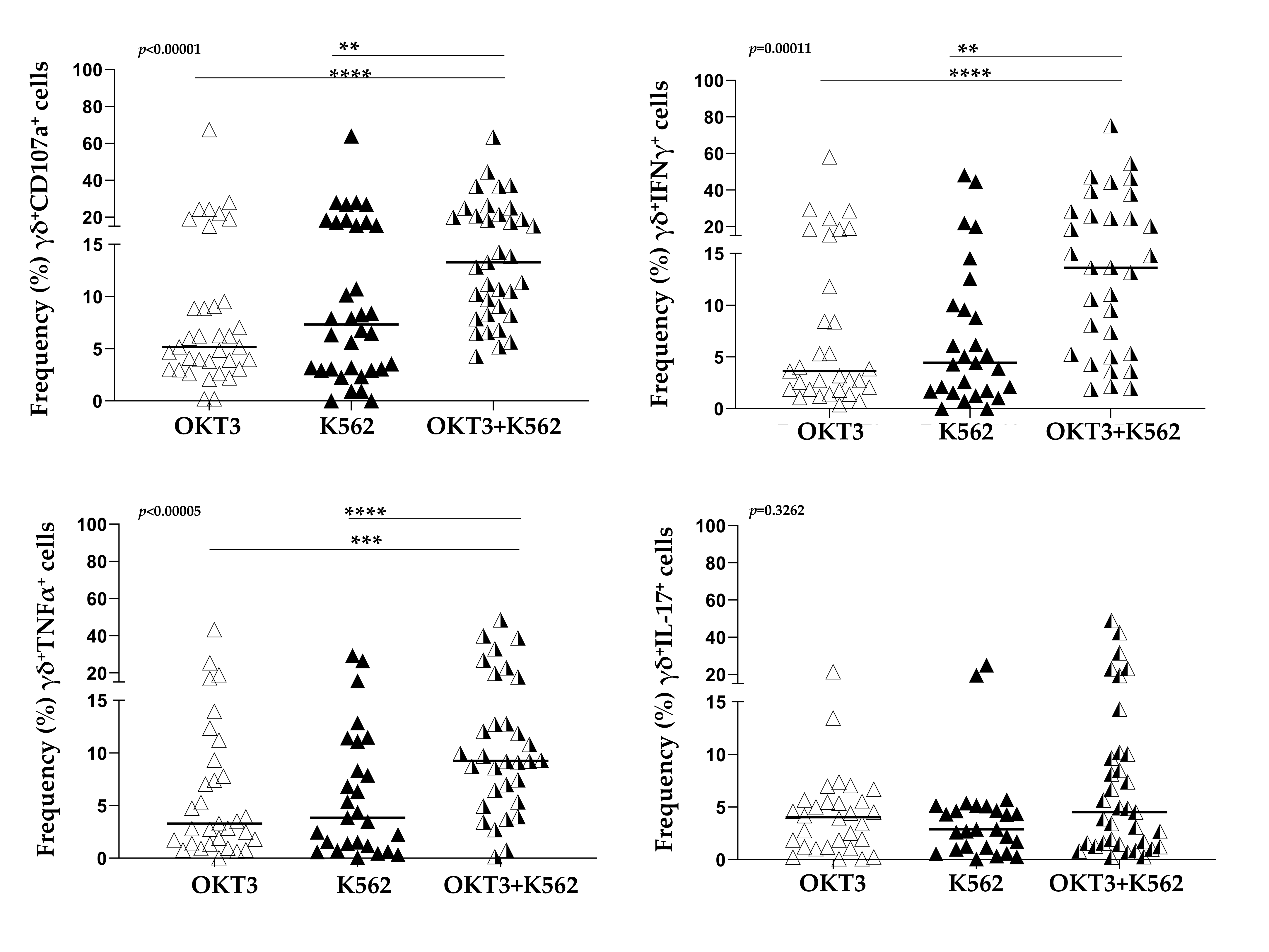

Supplement: Supplementary file 1 [file cells-15-01020-s001.zip › Figure S4.TIF]

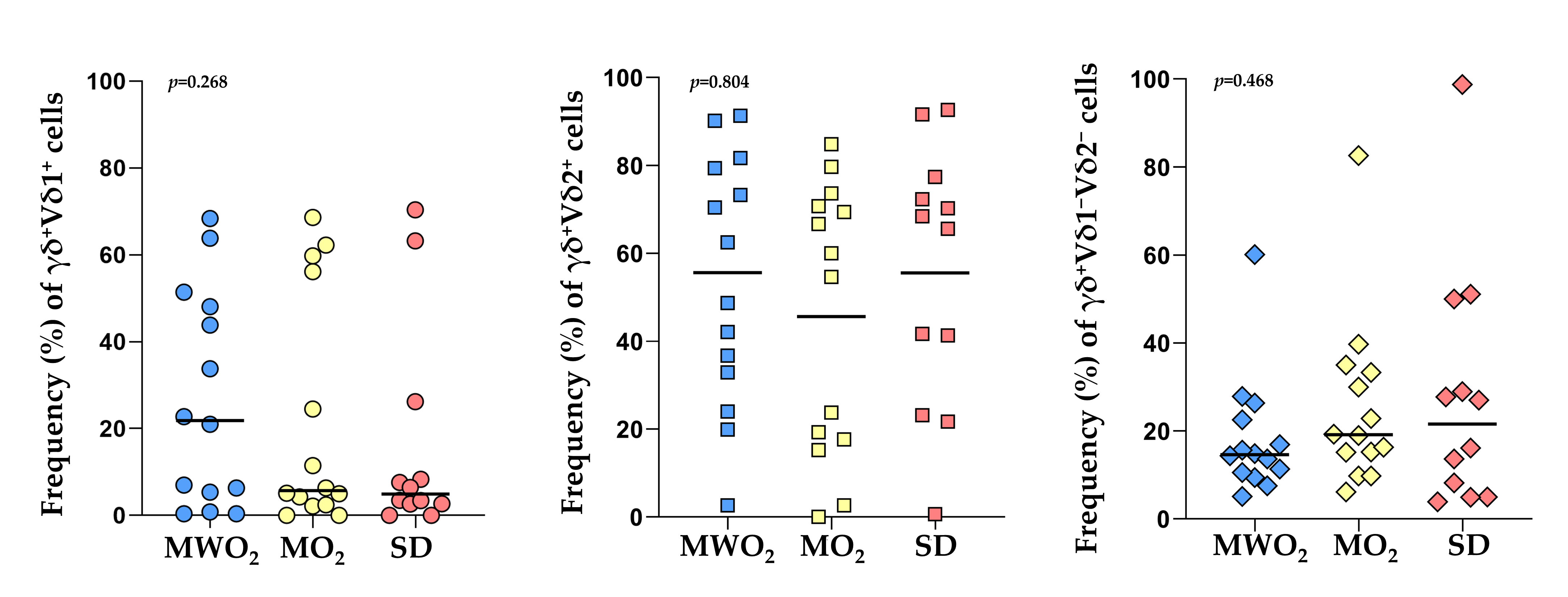

Supplement: Supplementary file 1 [file cells-15-01020-s001.zip › Figure S5.TIF]

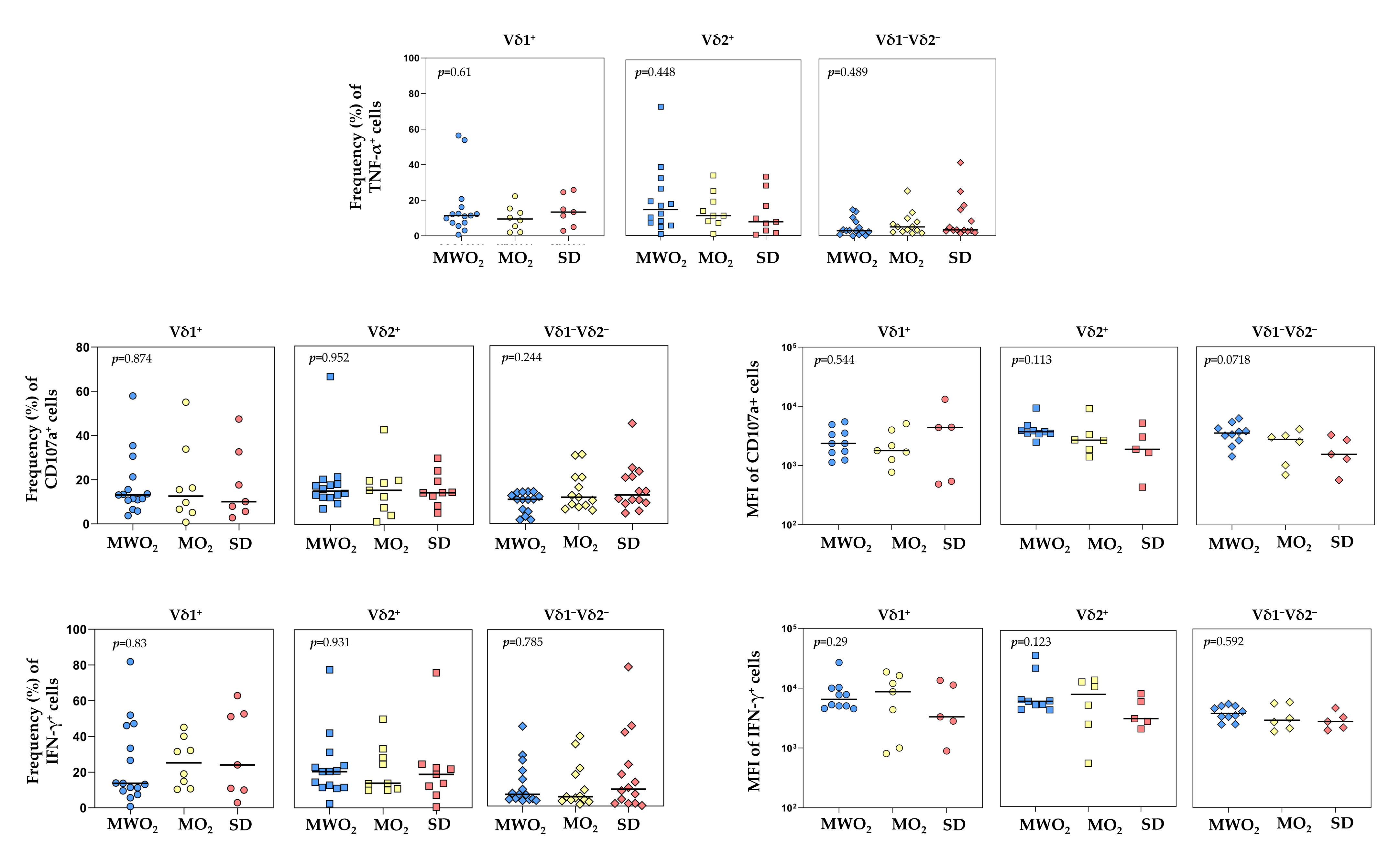

Supplement: Supplementary file 1 [file cells-15-01020-s001.zip › Figure S6.TIF]

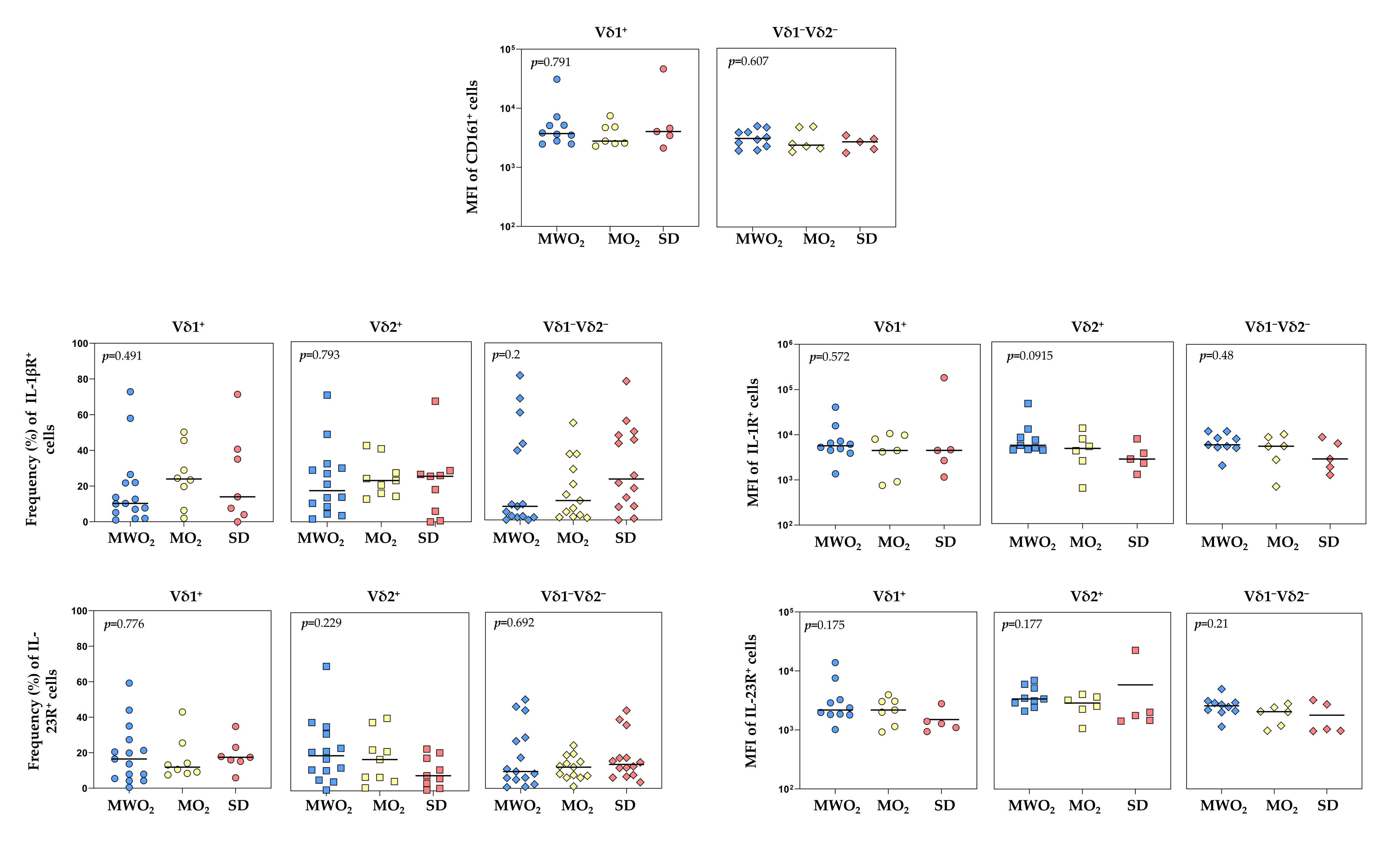

Supplement: Supplementary file 1 [file cells-15-01020-s001.zip › Figure S7.TIF]

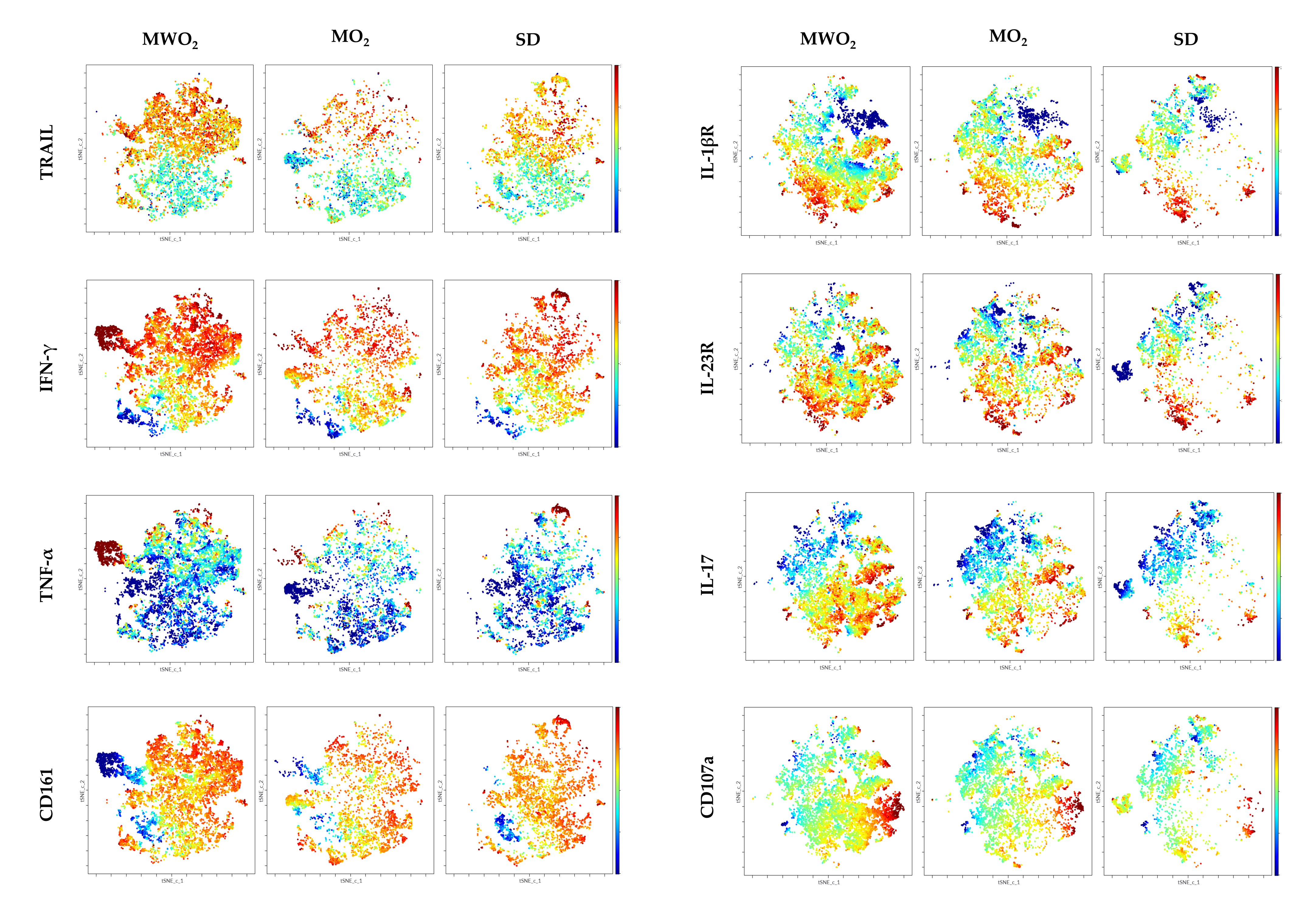

Supplement: Supplementary file 1 [file cells-15-01020-s001.zip › Figure S8.TIF]

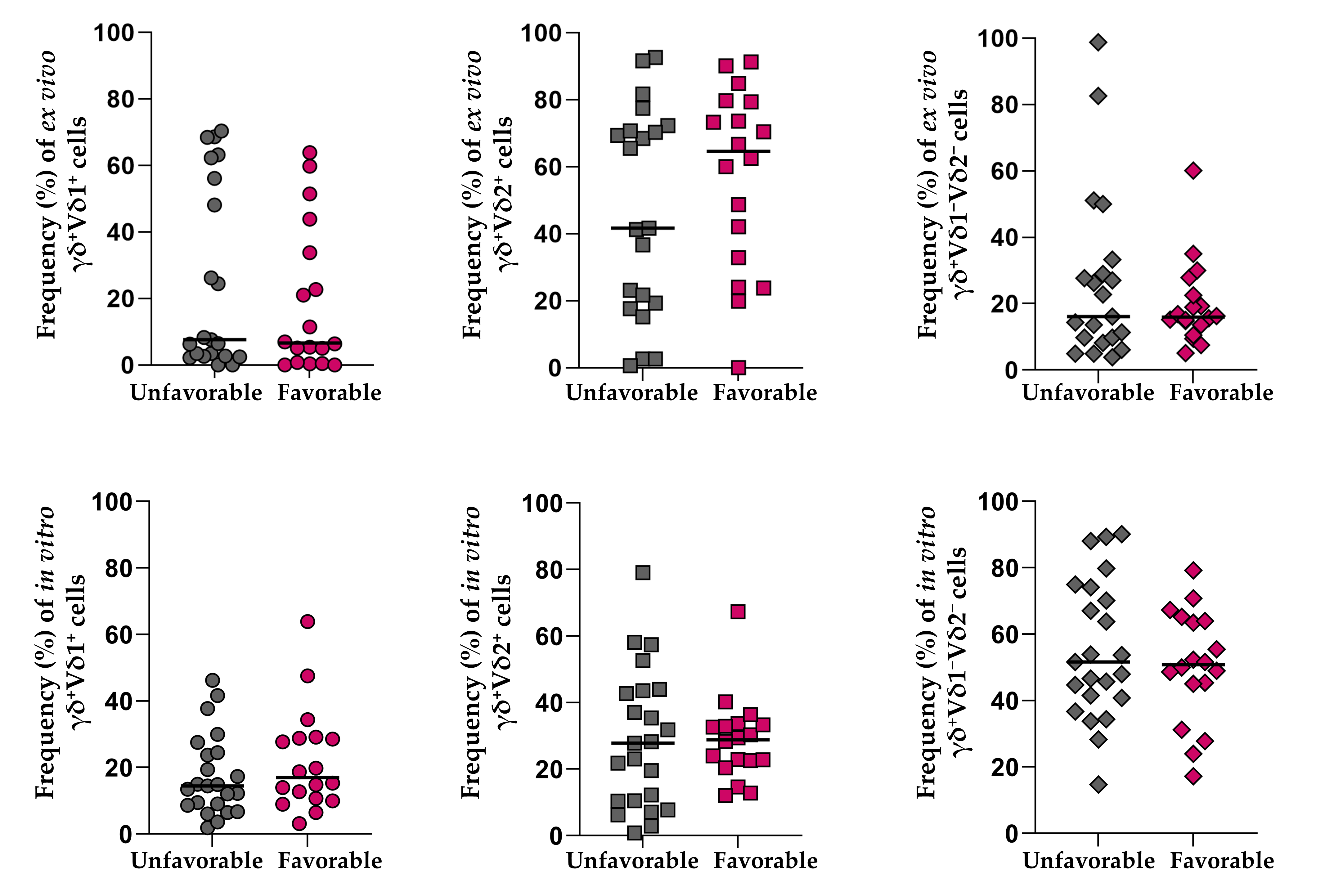

Supplement: Supplementary file 1 [file cells-15-01020-s001.zip › Figure S9.TIF]
